# Neutralising antibodies and protection from progression to severe COVID-19: A meta-analysis

**DOI:** 10.1371/journal.pmed.1005230

**Published:** 2026-09-02

**Authors:** Karen M. Elias, Ainslie Mitchell, Eva Stadler, Timothy E. Schlub, Shanchita R. Khan, Claire Xue, Stephen J. Kent, Deborah Cromer, Miles P. Davenport, David S. Khoury

**Affiliations:** 1 Kirby Institute, University of New South Wales, Sydney, Australia; 2 Faculty of Medicine and Health, Sydney School of Public Health, University of Sydney, Sydney, New South Wales, Australia; 3 School of Mathematics and Statistics, University of New South Wales, Sydney, Australia; 4 Department of Microbiology and Immunology, University of Melbourne at the Peter Doherty Institute for Infection and Immunity, Melbourne, Victoria, Australia; 5 Melbourne Sexual Health Centre and Department of Infectious Diseases, Alfred Hospital and Central Clinical School, Monash University, Melbourne, Victoria, Australia; Institut Pasteur, FRANCE

## Abstract

**Background:**

Vaccine-induced neutralising antibodies are a well-established correlate of protection against acquisition of COVID-19. Vaccine protection from severe COVID-19 remains high, although the role of neutralising antibodies and the contribution of other immune responses to protection from severe outcomes is less clear. Improving understanding of how vaccines prevent severe outcomes is important for future vaccine development.

**Methods and findings:**

Here we undertake a systematic search of the literature to obtain estimates of vaccine protection against progression from SARS-CoV-2 infection to severe COVID-19 (hospital or ICU admission). We aggregate results from 25 published clinical studies of vaccine protection from progression to severe outcomes in people with SARS-CoV-2 infection. We match this clinical data to data from 301 studies of post-vaccination neutralisation titres induced by various vaccines against different SARS-CoV-2 variants from the publicly available Stanford University Coronavirus antiviral and resistance database. We then synthesise the data using meta-regression. Neutralising antibody titres were found to be associated with vaccine protection against progression to severe COVID-19 (relative risk (RR) 0.57 per 10-fold increase in GMT, 95% CI [0.51,0.64], *p* < 0.001). We found that the protection against progression provided by vaccination was higher than that provided by therapeutic passive administration of antibodies, when used to treat individuals with confirmed infection. The main methodological limitations were the fact that neutralisation titres and clinical outcomes were measured in different studies, and the possibility of unmeasured confounding.

**Conclusions:**

Together, this work suggests that vaccine-induced neutralising antibodies are correlated with protection from progression to severe COVID-19 and can account for at least a partial mechanistic role in this protection. However, a protection gap remains between the high levels of protection against progressing to severe COVID-19 afforded by vaccination, compared to that afforded by therapeutic administration of antibodies, suggesting other immune responses may also play a role.

## Author summary

**Why was this study done?:**

- COVID-19 is globally endemic and remains a public health burden, especially in at-risk populations.
- This study aims to determine whether higher levels of neutralising antibodies are associated with greater protection from progression to severe COVID-19 in people who experience infection after vaccination (breakthrough infection).
- Understanding how vaccines help prevent severe outcomes is important for future vaccine development.

**What did the researchers do and find?:**

- Using a systematic search, we identified 25 studies that assessed the rates of disease progression from SARS-CoV-2 infection to severe COVID-19 comparing vaccinated and unvaccinated people.
- We found that vaccination significantly reduced the risk of people with SARS-Cov-2 infection developing severe outcomes.
- Matching these data with data on neutralising antibody titres after vaccination from 301 studies identified in a public database, we found that higher vaccine-induced neutralising antibody titres provided greater protection from progression to severe COVID-19.
- The protection afforded by vaccination against progression to severe COVID-19 was found to be higher than that afforded to unvaccinated individuals who received passive antibody treatments during COVID-19.

**What do these findings mean?:**

- Together, these results suggest that neutralising antibodies are correlated with protection against progression to severe COVID-19, and comparisons with passive antibody studies suggest that antibody responses alone are able to mediate at least some proportion of the protection against progression to severe COVID-19 achieved by vaccination.
- The gap in protection between passively administered antibodies and vaccination suggests the time from which neutralising antibodies are present during breakthrough infection and/or other immune responses, such as T cell responses and other nonneutralising antibody functions, are likely also involved in mediating protection against progression to severe COVID-19.
- An important limitation of this study is that the neutralising antibodies and rates of disease progression were not measured in the same individuals. There may be differences between these groups of people, which could not be accounted for during matching.

## Introduction

Vaccination is effective at preventing both symptomatic and severe COVID-19 [1–3]. The immune mechanisms that mediate protection against severe outcomes are a major focus of immunological investigation and improving our understanding of these processes has the potential to guide the development of next generation vaccines that better protect the most vulnerable. However, dissecting the immune responses that are centrally important or directly involved in protection against severe COVID-19 is difficult because correlations do not imply causation and the relatively low frequency of severe disease means large studies are required [4,5].

**Fig 1:**
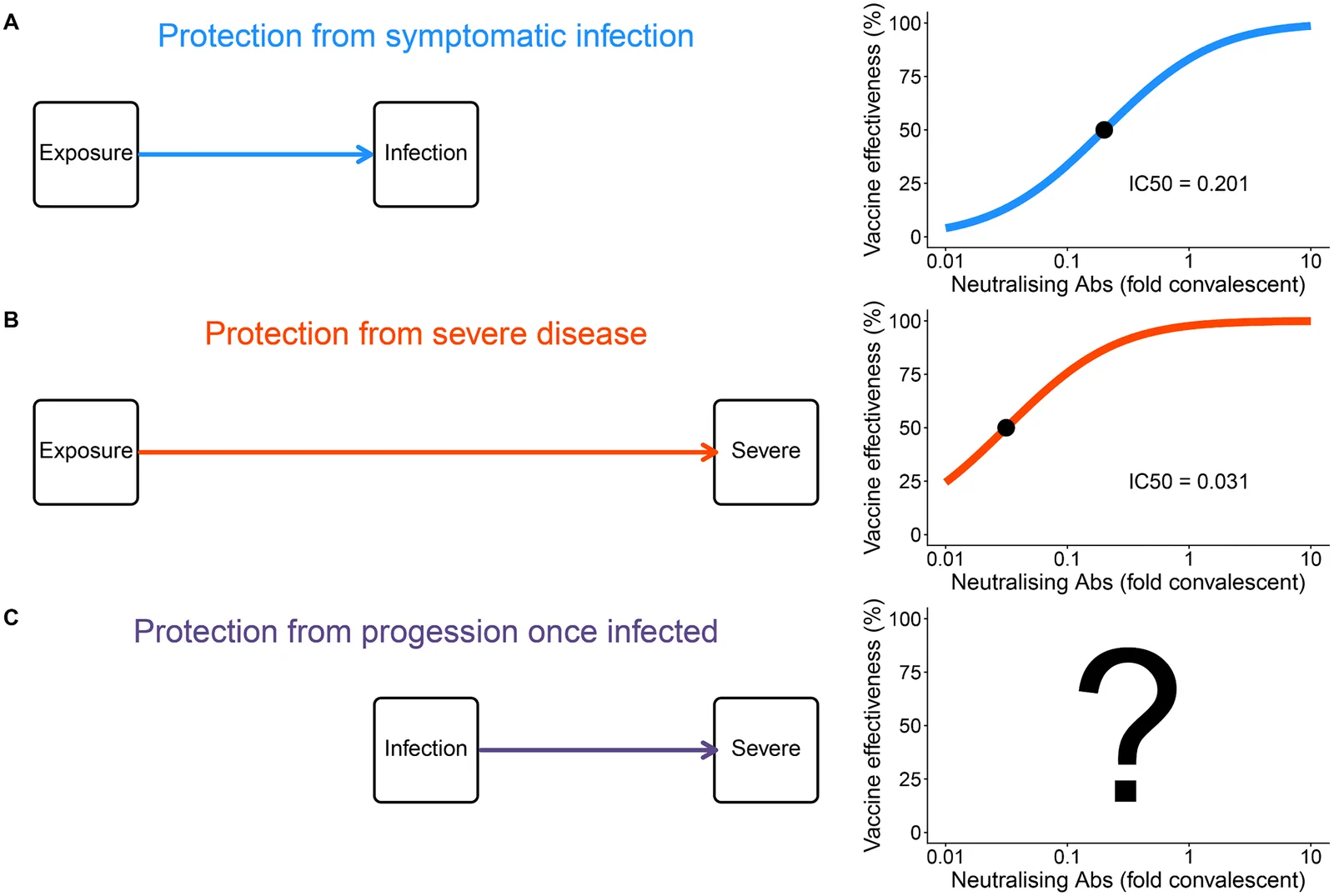
Relationship between neutralising antibodies and various measures of protection. The relationship between neutralising antibodies and protection from symptomatic infection **(A)**, and protection from severe outcomes **(B)** are reproduced from an existing analysis of RCTs of COVID-19 vaccines undertaken in 2021 [3]. Here we address the question of whether neutralising antibody titres are also associated with protection against progression to severe COVID-19 (**C**). The black dots show the IC50 for each type of protection.

Neutralising antibody titres are predictive of, and sufficient for, protection from symptomatic COVID-19 [3,6–8]. That is, neutralising antibodies both correlate with protection against COVID-19 [3,6,7], and antibodies alone (passively administered as prophylaxis in otherwise naïve individuals) achieve protection against acquisition of COVID-19 at similar antibody levels to that seen in active vaccination [8,9]. In the case of severe COVID-19, neutralising antibodies have also been shown to correlate with protection [10,11]. However, since acquiring severe COVD-19 requires first acquiring symptomatic COVID-19, it is perhaps not surprising that neutralising antibodies correlate with protection against severe disease, given they correlate with protection against mild disease (Fig 1A and 1B). More relevant to our understanding of immunity against severe COVID-19 is understanding the amount of vaccine-induced protection against *“*progression*”* to severe COVID-19 if a breakthrough infection occurs (Fig 1C). That is, if vaccination fails to prevent infection (which often occurs), how well does the vaccine protect people with mild infection from progressing to a more severe event (Fig 1C)? Furthermore, is this vaccine-conferred protection against “progression” also correlated with neutralising antibodies (i.e., independent of their role in protection against acquiring mild COVID-19) (Fig 1A)?

In this study, we aim to determine whether vaccine-induced protection against progression to severe COVID-19 is associated with neutralising antibody titres. We first performed a systematic search and meta-analysis of clinical studies investigating the risk of progression to severe outcomes comparing vaccinated and unvaccinated individuals. We then tested if this protection is correlated with the neutralising antibody responses induced by vaccination. Finally, we compared the level of protection against progression to severe COVID-19 provided by active vaccination and therapeutic passive antibody administration to see if these are comparable, or if active vaccination provides greater protection than passively transferred antibodies alone, i.e., whether vaccine-induced immune responses other than neutralising antibodies also contribute to protection against progression.

## Methods

### Ethics statement

This work was approved under the UNSW Sydney Human Research Ethics Committee (approval HC200242). Individual participant informed consent was not required for this study as it was an analysis of previously published data. There was no direct interaction with human participants, and no new data was collected from human participants.

### Vaccine protection against progression from SARS-CoV-2 infection to severe COVID-19

In order to find evidence of vaccine protection against disease progression we searched PubMed for all studies published in English from 01 Dec 2020 to 23 Jan 2025 which assessed outcomes in a population who were already infected with SARS-CoV-2 and where rates of progression to severe COVID-19 were compared between vaccinated and unvaccinated people. We excluded studies that did not compare outcomes in vaccinated versus unvaccinated populations (e.g., studies were excluded if they only compared boosted individuals to those with a primary course, or compared one vaccine to another), and studies which recruited from emergency department presentations, since it is likely that individuals presenting at emergency departments may have already been progressing to more severe symptoms (see Supplementary materials in S1 Appendix for full search strategy).

For each of the clinical studies, we extracted data on administered vaccine, number of doses received, infecting SARS-CoV-2 variant, rates of prior infection, the clinical outcome used to define progression to severe outcomes, and the statistical risk measure (e.g. aOR, aHR, etc.) used to compare the risk of progression between the vaccinated and unvaccinated groups. Where the effect of vaccination on disease progression was disaggregated by the vaccine received, this data was extracted. Where the effect of vaccination was not disaggregated by vaccine in a paper, we firstly treated different mRNA vaccines (i.e., BNT162b2 and mRNA-1273) as equivalent, and whole inactivated vaccines (i.e., CoronaVac and BBIBP-CorV) as equivalent, then we recorded the most common vaccine type received by participants (or excluded the study if the most common vaccine type was received by less than 50% of the vaccinated population). Each group of vaccinated individuals included in the study was categorised as having received a particular vaccine regimen which was defined as a combination of vaccine type and the number of doses received by the individuals. Where vaccine protection was reported against single SARS-CoV-2 variants, this data was extracted. If protection against a single variant was not available, combined variants were used, noting that any studies that combined vaccine effect across pre-Omicron and Omicron variants were excluded (according to our exclusion criteria). Further details are provided in the Supplementary materials in S1 Appendix.

### Neutralising antibodies

To obtain data on neutralising antibody titres after vaccination for a variety of vaccine regimens we used the Stanford University Coronavirus antiviral and resistance database [12]. This database contains post-vaccination in vitro neutralisation titres from studies that were comparing neutralisation between variants using a variety of assays. We searched the database for neutralisation titres in people with no prior infection against the ancestral strain, or variants present in the clinical studies (i.e., Alpha, Beta, Delta, Omicron BA.1, Omicron BA.2, Omicron BA.4, and Omicron BA.5), and for each of the vaccines present in the clinical studies (see Supplementary materials in S1 Appendix), resulting in data from 301 underlying immunological studies (2,156 separate titre estimates). We performed a meta-analysis of the neutralising antibody data from this database using a linear mixed-effects model (using the lmer function from the lme4 package in R) [13], with random intercepts for study (see Supplementary materials in S1 Appendix). This produced global estimates of the neutralisation titre after vaccination with different vaccine regimens, against different variants and at different times after vaccination (for example, we obtained a global estimate of the neutralisation titre to Omicron BA.1 one month after 3 doses of BNT162b2).

### Linking neutralising antibody levels and disease progression data

The neutralising antibody titres obtained from the meta-analysis above were then matched to the clinical data on vaccine protection from progression to severe COVID-19. Matching was performed based on: vaccine regimen, SARS-CoV-2 variant, and time since last dose where available. Where the clinical data that was reported could be matched to more than one category of neutralisation data, we matched it to the geometric mean of the relevant neutralisation titre categories (e.g., if the clinical data reported outcomes for a population who were infected with either Omicron BA.1 or Omicron BA.2, then the geometric mean of the GMTs for the relevant variants was used). The full details of matching of vaccine effectiveness studies to results of the neutralisation meta-analysis are described in the Supplementary materials. Once matching was completed, we analysed the relationship between vaccine-induced neutralising antibody titre and protection from progression to severe disease (see Statistical methods for details).

We have previously analysed the association between vaccine-induced neutralising antibody titre and protection from mild and severe COVID-19 [3]. Here we have used these relationships to model protection against progression from mild to severe infection as a validation step. Similarly, we have previously analysed studies of passive antibody administration as a treatment for COVID-19, early after symptom onset, and found a relationship between the administered dose (neutralising antibody titres) of passively administered antibodies and protection against progression to severe outcomes [14]. To allow comparison with these prior works, we normalised the estimated neutralising antibody titres used in our study (from the Stanford database) [12] to a scale of “fold-convalescent” also using data obtained from the Stanford database [12]. We extracted (from the Stanford database) the aggregate geometric mean neutralisation titre (against ancestral SARS-CoV-2) in convalescent individuals at both 1 month and 2−6 months post infection with ancestral SARS-CoV-2. The geometric mean of these two time periods (indicated with a dashed line in Fig E in S1 Appendix) was then used to normalise other neutralisation titre estimate to a fold-convalescent scale (i.e., by dividing a GMT by this convalescent titre). This fold-convalescent scaling approach was chosen in order to best align with the scaling approach we employed previously in Khoury and colleagues [3].

### Statistical methods

Protection from progression was reported in the primary clinical studies using a variety of statistical risk measures (e.g., relative risk (RR), odds ratio, hazard ratio), which all converge when events (progression to severe outcomes in this study) are rare [15]. These estimates of protection were aggregated using an inverse variance weighted multilevel linear mixed-effects model (using the rma.mv function with maximum likelihood estimation from the metafor package in R) [16]. Vaccine protection was expressed as the natural log-transformed RR, and variance in these protection estimates was calculated from the 95% confidence intervals published in the underlying studies. For the random-effects structure, we used random intercepts at the study level (Level 3) and at the effect-size-within-study level (nested at Level 2), reflecting the nonindependence of multiple effect sizes per study. This model was compared to the simpler model, with between-study clustering only, using likelihood ratio tests (with restricted maximum likelihood fitting, see Supplementary materials in S1 Appendix). More complex random effects structures were not explored. Candidate covariates were identified by clinical relevance and data availability. We evaluated all possible combinations of the candidate covariates (see Supplementary materials in S1 Appendix), excluding interactions between them, and compared the models using AICc (Akaike information criterion with correction for small sample sizes) from the MuMIn package in R [17].

The matched neutralisation titres were then used in a univariate meta-regression by including log_10_(GMT) as the only fixed-effect covariate in a model with the same multi-level random effects structure described previously. To test the robustness of this relationship we investigated whether this relationship was sensitive to the inclusion of additional covariates in multivariate analyses. Statistical significance of covariates was calculated using likelihood ratio tests. Ninety-five% Confidence intervals for all meta-regression model outputs were calculated as 1.96 times the standard error, and the 95% confidence regions on regression lines were calculated using the predict() function in R.

The reported estimates of vaccine protection against progression, from the underlying clinical studies, were visualised using Forest plots. Funnel plots were used to assess potential publication bias.

All correlations were assessed using a Spearman correlation. All statistical analyses were performed in R version 4.2.1, with a threshold for statistical significance of 0.05.

## Results

### Vaccine protection against progression from SARS-CoV-2 infection to severe COVID-19

We first aimed to quantify vaccine protection against progression to severe COVID-19. Thus, we searched PubMed for studies that contained data on the risk of progressing from SARS-CoV-2 infection to severe COVID-19, comparing vaccinated versus unvaccinated individuals so that we could estimate the protection afforded by vaccination (see Supplementary materials in S1 Appendix for full search strategy). Our search identified 2,280 unique records of which 25 [18–42] met our inclusion criteria (Supplementary materials, Tables A-B, Fig A in S1 Appendix). We extracted data on administered vaccines (including type, number of doses, and timing of last dose where available), SARS-CoV-2 variant (typed at infection or reported to be the dominant circulating variant at the time of the study), and reported results relating to vaccine protection against disease progression. This was done using the most disaggregated data (based on vaccine, number of doses, variant and time since last dose) available from each study, leading to a total of 137 estimates of vaccine protection against progression to severe outcomes being extracted from the 25 studies, with individual studies contributing anywhere from 1 [18–25] to 40 [36] estimates of vaccine protection. The studies reported a variety of different clinical endpoints, with some studies reporting on a single endpoint, while others reported results separately for multiple clinical endpoints. The most commonly available endpoint was hospital admission (20/25 studies, 130/137 observations), so our main analysis used this endpoint where it was available and used ICU admission in the remaining five studies (7/137 observations). The proportion of individuals in each study who had a history of prior infection was 0% in 5/25 studies, <10% in 7/25 studies, 10%–20% in 1/25 studies and was not reported in 12/25 studies (Table B in S1 Appendix). Importantly, our analysis always focussed on protection in vaccinated individuals (who may have received 1 dose of Ad26.COV2.S, or 2, 3 or 4 doses of all other vaccines) compared to unvaccinated individuals in the same study. The vaccine protection from progression reported in all underlying studies has been summarised in Forest plots (Figs B-C in S1 Appendix). Across all included data (pooling different vaccines, variants, number of doses, time since last dose, etc) the observed vaccine protection from progression to severe COVID-19 was 73% (RR 0.27, 95% CI [0.21,0.33], *p* < 0.001) (Table G in S1 Appendix).

### Assessing predictors of vaccine protection against progression

**Fig 2:**
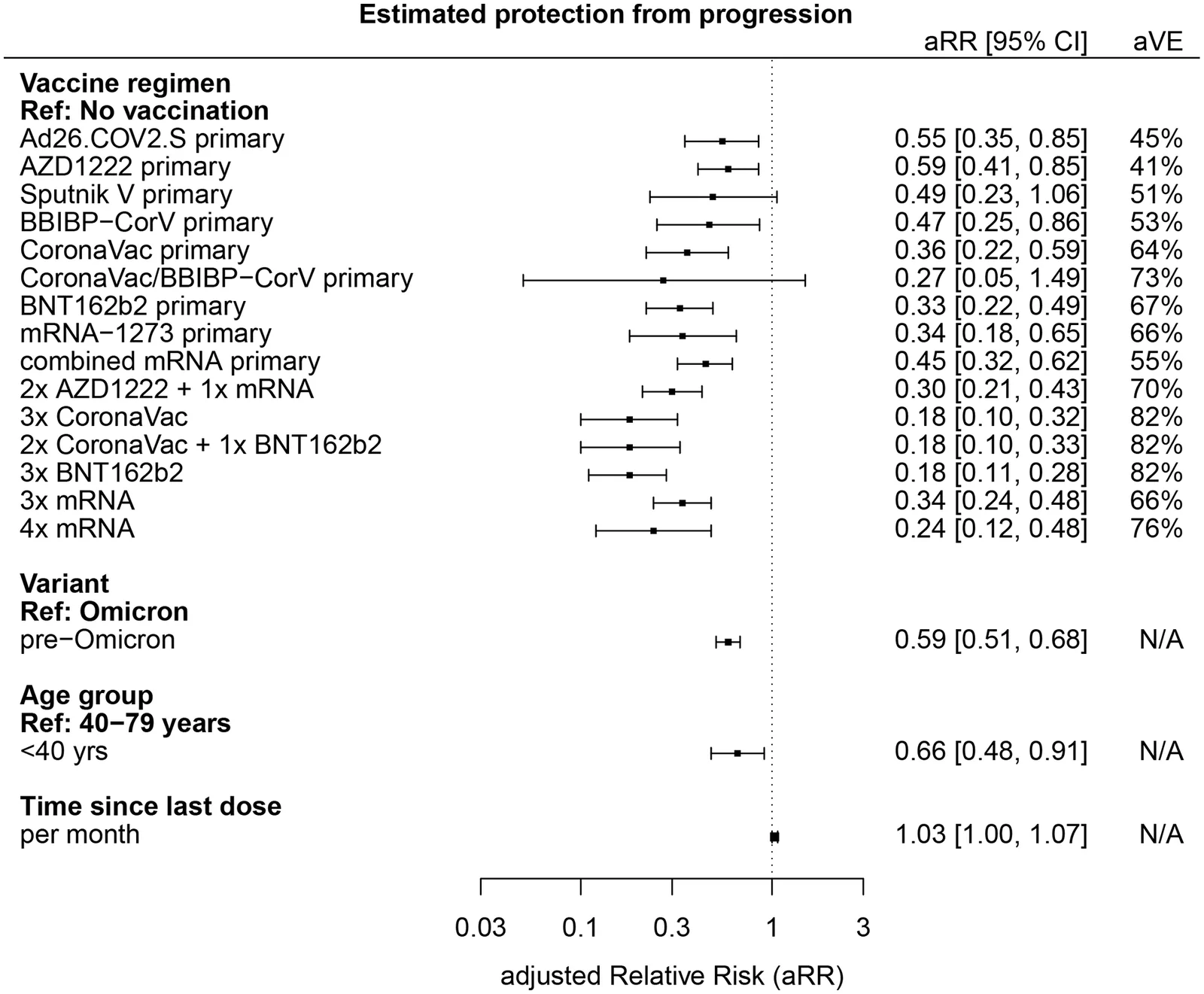
Predictors of vaccine protection against progression to severe outcomes. Points and lines denote estimates and their 95% confidence intervals for the adjusted relative risk (aRR) of the covariates in the model of vaccine protection. The dotted line is a relative risk of 1 (i.e., equivalent to risk in the reference group). Values lower than 1 (to the left of the dotted line) occur where risk in the stated group is lower than the reference group, while those greater than 1 have higher risk than the reference group. For clarity we also report the corresponding adjusted Vaccine effectiveness (aVE) for each vaccine regimen. aVE is calculated as 100 x (1 – aRR) and is reported here against the Omicron variant in the 40–79 years age group as at day of most recent dose (Table I, Fig D in S1 Appendix). N/A, Not applicable.

We expected vaccine protection against progression may differ by factors such as vaccine regimen, infecting variant, and days post-vaccination. Therefore, to account for these potential factors, as well as other potential confounders, we considered all candidate covariates (see Supplementary materials in S1 Appendix) and compared models containing all possible combinations of these using AICc. The model with the lowest AICc included vaccine regimen, variant, time since last dose and age group (<40 years or 40−79 years) as covariates (Forest plot Fig 2, Tables H-I in S1 Appendix). We observed from this multivariate model that vaccine protection against progression was higher after a third or fourth dose (when compared to unvaccinated individuals) than it was after a primary course only (e.g., 2 dose BNT162b2 versus unvaccinated (RR 0.33, 95% CI [0.22,0.49]), 3 dose BNT162b2 versus unvaccinated (RR 0.18, 95% CI [0.11,0.28])). Protection against progression was also higher against pre-Omicron variants than Omicron variants (RR 0.59, 95% CI [0.51,0.68]), and higher in younger cohorts (RR 0.66, 95% CI [0.48,0.91]) for mean/median age <40 yrs compared to 40−79 years (Fig 2, Table I and Fig D in S1 Appendix). Together, this provides evidence that vaccines are effective at preventing COVID-19 progressing to severe COVID-19 and there is some evidence that this differs by age, SARS-CoV-2 variant, time since last dose and vaccine regimen.

### Relationship between neutralisation titres and protection against progression

We next aimed to determine if vaccine protection against progression to severe COVID-19 is associated with neutralising antibody titres after vaccination. Since neutralising antibody titres were not measured in the clinical studies themselves, we linked each estimate of vaccine protection from progression in the clinical studies to an estimate of the geometric mean neutralising antibody titre in a matched population of vaccinated individuals [12] (see Table 1, Tables C–F and Fig E in S1 Appendix). Matching was based on vaccine regimen (vaccine and number of doses), variant, and time since last dose. This then allowed us to test whether neutralising antibody titres were associated with vaccine protection from progression to severe disease.

**Fig 3:**
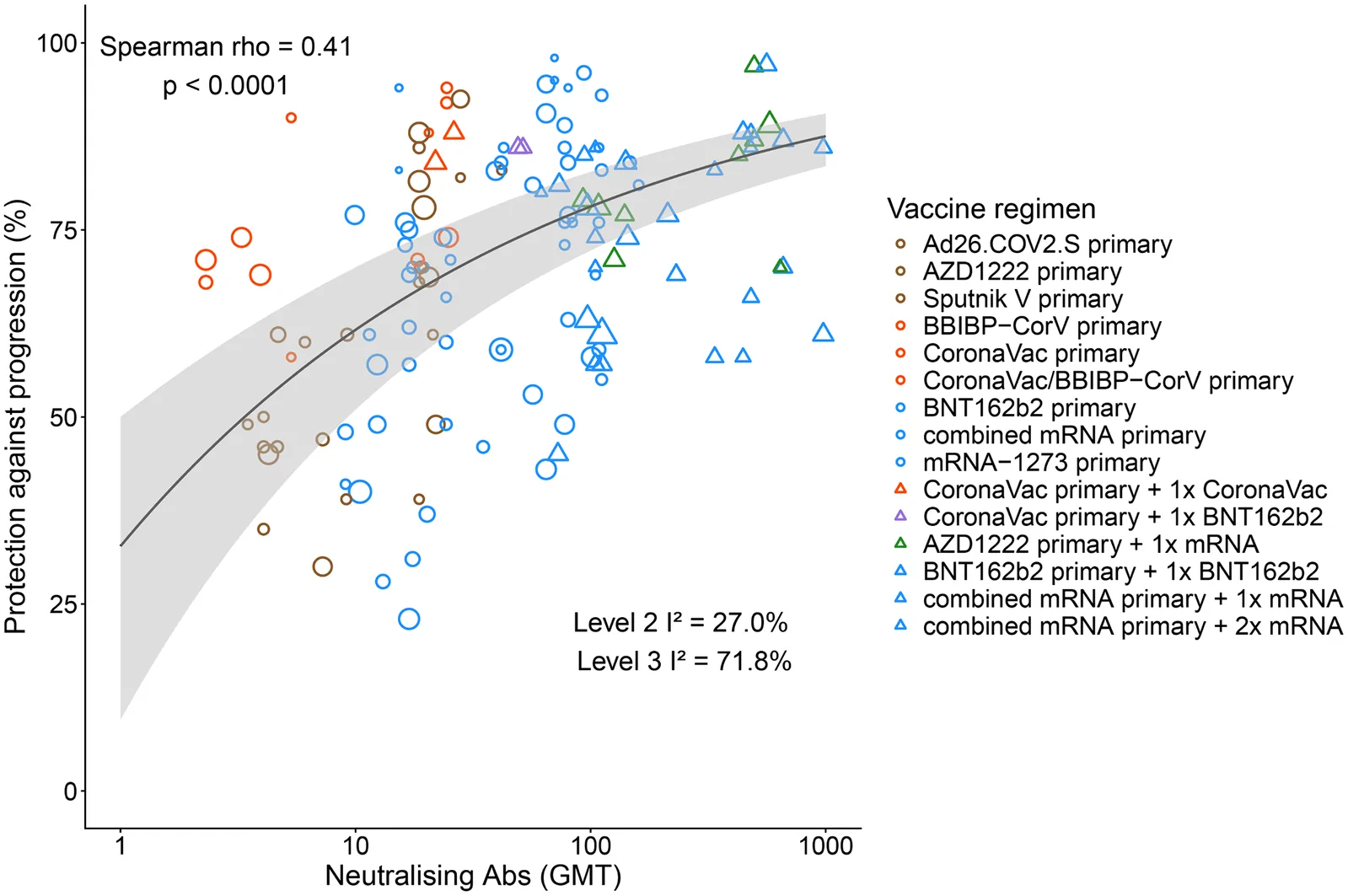
Relationship between (geometric mean) neutralisation titre and vaccine protection against progression to severe outcomes. Here we show on the *x* axis, the geometric mean neutralisation titre (GMT) that was matched to each reported result of protection (vaccine effectiveness) against progression to severe diseases (shown on *y* axis). Protection against progression is calculated as 100 x (1 – aRR), where aRR is adjusted relative risk. Shapes represent vaccination levels (circles for primary course, triangles for groups with 3 or more doses). Colours show vaccine type, and larger point sizes indicate protection estimates with narrower confidence intervals. The black line is the fitted model from our univariate meta-regression model of neutralisation titre and protection (i.e., no additional covariates for variants or ages). The grey shaded region shows the 95% CI.

We found a significant correlation between matched neutralisation titres and protection from disease progression (Spearman r = 0.41, *p* < 0.001, Fig 3). Furthermore, in a univariate meta-regression analysis, neutralisation titres were found to be a significant predictor of vaccine protection against progression (RR 0.57 per 10-fold increase in GMT, 95% CI [0.51,0.64], *p* < 0.001), (Fig 3, Table J in S1 Appendix).

To test the robustness of this association, we considered whether the association remained after adjusting for additional covariates, including vaccine regimen, variant, time since last dose and age group. Neutralising antibodies remained significantly associated with vaccine protection regardless of whether any one of these additional covariates was added to the model (Table K in S1 Appendix). Interestingly, we also noted that vaccine regimen and time since last dose were no longer significant covariates once neutralising antibody titres were included in the model (LRT *p* = 0.075 and *p* = 0.92, respectively, Table K in S1 Appendix). This suggests that differences in vaccine protection between regimens (and time since last dose) were largely explained by neutralising antibody titres in this meta-analysis. Age group (<40 versus ≥40 yrs) and variant (Omicron versus pre-Omicron) remained significant covariates along with neutralising antibody titres (LRT, *p* = 0.016 and *p* = 0.011 respectively, Table K and Fig F in S1 Appendix). Even so, in these models, as well as in a model containing all three predictors (age group, variant, and neutralising antibody titre) neutralising antibodies remained significantly associated with protection against progression (Supplementary materials and Table L in S1 Appendix). Together, suggesting that the association between neutralising antibodies titres and protection against progression observed here is robust to the inclusion of potential covariates.

To test the robustness of the association between neutralising antibody titres and protection against progression, we also performed a series of additional sensitivity analyses (see supplementary results, Tables M-N and Figs G-I in S1 Appendix). These included repeating the analysis with COVID-19 related death as an outcome (Table M in S1 Appendix), and the influence of individual studies on the results (Figs G-H in S1 Appendix). We noted that four studies had rates of progression >10% and we conducted a sensitivity analysis excluding these four studies which showed consistent results (see supplementary results, Tables B, M in S1 Appendix). In order to assess whether the inclusion of studies with either unknown or high (>10%) rates of prior infection was impacting our results, we ran a sensitivity analysis excluding these studies, and found no changes to our conclusions (Table M in S1 Appendix). In all cases, neutralising antibody titres remained a significant predictor of protection from progression. Visual inspection of funnel plots showed some asymmetry (Fig I in S1 Appendix), suggesting the possibility of publication bias; however, it is likely that the asymmetry is at least partly due to study heterogeneity given the high between-study variability (Level 3 I^2^ = 71.8%).

Together, our analyses suggest that neutralising antibodies are associated with protection from progression to severe outcomes, and that this relationship is robust despite study differences and after adjustment for potential confounders.

### An external validation of the relationship between neutralising antibody titres and protection from progression

**Fig 4:**
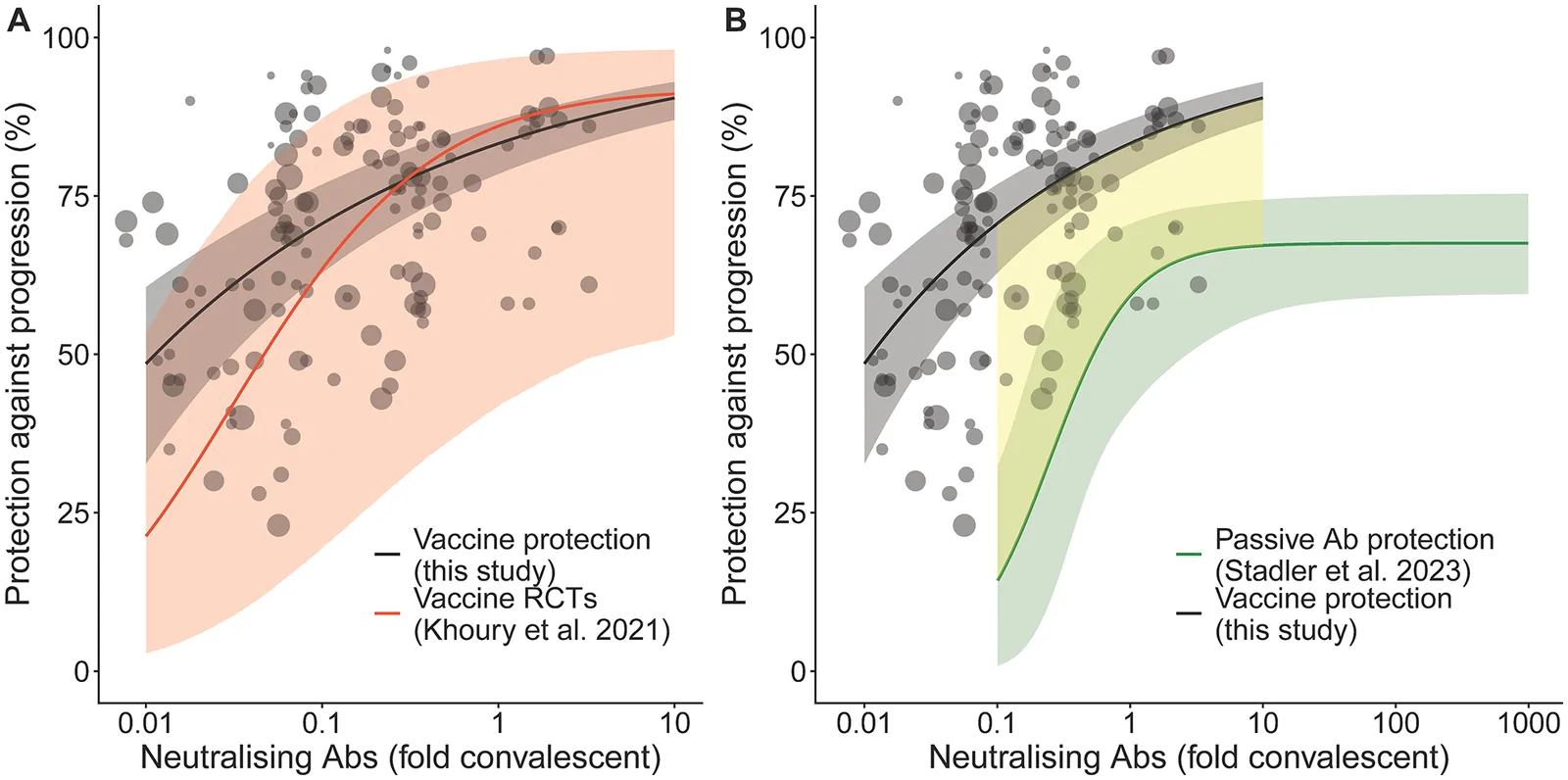
Comparison with previously determined relationships between antibody titre and protection. **(A)** The red line shows the predicted relationship between vaccine protection against progression to severe outcomes and neutralisation titre (which was derived from the symptomatic and severe relationships predicted from an analysis of RCTs (randomised controlled trials) of COVID-19 vaccines in 2021 [3], see Supplementary materials in S1 Appendix). The black line shows the relationship between vaccine protection and neutralisation titres derived here (same as Fig 3, with the scale on the x-axis converted to fold-convalescent – see supplementary material). Shaded regions are 95% CIs. **(B)** Comparison of protection against progression to severe disease seen between vaccination and therapeutic antibody administration. The green line shows the previously determined relationship between neutralisation titres and protection from progression in those with COVID-19 who received therapeutically administered antibodies [14], while the black line shows the meta-regression model developed here (same as Panel A). The yellow shaded region shows the “protection gap” between the two associations, for the common range of neutralising antibodies. Notice that for any given level of neutralising antibody titres, protection against progression to severe disease is higher from vaccination than passively administered antibodies alone. Protection against progression is calculated as 100 x (1 – RR), where RR is relative risk.

To assess whether the relationship between neutralisation and vaccine protection against progression found here is consistent with prior work, we compared it with the relationship predicted from an analysis of randomised controlled trials of COVID-19 vaccines (in March 2021) [3,43]. This relationship reported by Khoury and colleagues also used a meta-analysis and was not focussed on the outcome of progression to severe outcomes and had limited power due to the low event counts for severe outcomes. However, the study quantified the relationship between neutralisation titres and protection against both symptomatic and severe COVID-19 (Fig 1A and 1B respectively). In that study, at any given neutralisation titre, the protection against severe outcomes was higher than the protection against symptomatic infection. For any neutralisation titre, the (multiplicative) difference between these curves represents the model’s predicted vaccine protection against progression to severe disease (Fig 1C, Supplementary materials in S1 Appendix). After converting the neutralisation titres in the above meta-analysis to a fold-convalescent titre to make them comparable with Khoury and colleagues, we find that the relationship identified here between neutralisation titres and protection from progression to severe disease lies within the confidence interval of the predicted relationship derived in March 2021, based on comparing vaccine protection from mild versus severe COVID-19 from the vaccine RCTs (Fig 4A). This concordance highlights that two independent studies, using different data, from different underlying study types and different methods arrive at very similar relationships between neutralisation titres and protection from progression to severe COVID-19, providing some support for the estimated association.

### Considering neutralising antibodies as mechanistic in protection against progression to severe outcomes

In the above, we observe an association between neutralising antibody titres and vaccine protection against progression to severe COVID-19. However, it remains to be determined whether neutralising antibodies are mechanistic in preventing progression to severe outcomes or merely a surrogate biomarker of overall immune response magnitude [44]. Studies on the therapeutic use of passively administered antibodies (both monoclonal antibodies and convalescent plasma) to prevent progression to severe COVID-19 [14] provide an opportunity to directly assess the mechanistic role of antibodies in preventing progression.

Previously, in a meta-analysis of passive antibody treatments, we have shown a dose response relationship between the administered dose of passive antibodies (normalised by in vitro neutralisation titre/IC50) and their effectiveness at preventing progression to severe outcomes [14]. We compared the protection against progression from vaccination (estimated above) and that observed after the therapeutic use of passive antibodies at similar neutralising antibody titres (see Supplementary materials in S1 Appendix) and found that protection against progression to severe disease is indeed lower in passive antibody administration than it is for vaccination for any given neutralising antibody titre (Fig 4B). This suggests there is a “protection gap” between the protection that can be achieved with passively administered antibodies during early infection and vaccine protection for a given vaccine-induced neutralising antibody titre. We quantified this protection gap as an approximately 63% lower risk (RR = 0.37, 95% CI [0.24, 0.55]) of progression to severe outcomes with active vaccination (average across neutralising antibody titres, see Supplementary materials in S1 Appendix), compared to passive antibodies. This quantity represents the average additional protection observed after active vaccination compared to after passive antibody administration for the range of neutralising antibody titres common to both passive antibody treatments and vaccination.

There are several fundamental differences between vaccination and passive antibody therapy (and especially monoclonal antibody therapy) that could explain this protection gap. Firstly, in vaccinated individuals, antibodies will be present prior to infection, while for those receiving passive antibody treatment, antibodies are generally administered 2–5 days after symptom onset [14]. Secondly, vaccination induces a more diverse immune response, including polyclonal antibodies, multiple antibody isotypes, T-cells and other cellular immune responses (all or some of which may provide additional protection not seen when administering passive antibody treatments). Finally, active vaccination produces memory responses that are capable of recall. Thus, the antibody titres measured prior to infection may not be reflective of the level of antibodies present during infection. These differences lead us to expect lower effectiveness of passive antibody treatments compared to vaccination.

A number of previous studies have shown that administering therapeutic antibodies earlier after COVID-19 symptom onset yields higher protection from progression to severe outcomes [14,45,46]. We previously estimated the relationship between time-of-administration of passive antibody (post-symptom onset) and protection from severe outcomes (See Fig S11 and Table S6 from Stadler *et al*. [14]). In that study, we estimated that if antibody administration had occurred on the day of symptom onset, then the risk of progression would be approximately 52% lower (RR 0.48, 95% CI [0.28, 0.82]) compared to the actual time of administration in the studies analysed (which occurred an average of 4.2 days after symptom onset). The level of increased protection between active vaccination and passive antibody administration (63% lower risk of progression in vaccination than passive antibody treatment) is very similar to the increased protection expected if antibodies were given at symptom onset. This suggests that most of the protection gap between passive antibody therapy and active vaccination (~77%, Fig J in S1 Appendix) might be explained by passive antibodies only being administered several days after symptom onset rather than being present since the time of infection (as is the case in active vaccination).

## Discussion

There are many studies showing an association between vaccine-induced immune responses and protection from symptomatic COVID-19. However, demonstrating the impact of vaccination in preventing the development of severe COVID-19 is challenging. Here we aggregate data from 25 clinical studies on progression from SARS-CoV-2 infection to severe COVID-19 and 301 studies on antibody titres after vaccination, to examine the relationship between neutralising antibody titres and protection from progression. We find that vaccine-induced neutralising antibody titres are consistently associated with vaccine-mediated protection against progression from SARS-CoV-2 infection to severe COVID-19. This was the case across both Omicron and pre-Omicron variants, and despite substantial heterogeneity between the included studies. This relationship is very similar to the relationship derived previously from analysis of vaccine protection data from clinical RCTs [3], despite the very different study types.

We also sought to understand the degree to which neutralising antibodies might be mechanistic in protection from developing severe COVID-19, rather than just correlated with protection. We compared the protection against progression to severe COVID-19 achieved by vaccination with that achieved by passive antibody administration to infected individuals (who were otherwise naïve) (Fig 4B) [14]. We observe that for any given level of neutralising antibodies, protection against progression was higher from active vaccination than from passive antibody therapy (with 63% lower risk compared to those who received passive antibodies). This “protection gap” may indicate that other nonserological immune responses, such as vaccine-induced T-cells, contribute additional protection from progression to severe outcomes over and above the neutralising antibodies. However, another possibility is that passive antibodies, having been administered on average 4.2 days after symptom onset in the relevant trials, may not be as effective at preventing severe COVID-19 as neutralising antibodies present prior to infection (as in active vaccination). In support of the latter hypothesis, we have previously estimated that passive administration of antibodies could provide as much as 52% more protection from progression if given immediately after symptom onset rather than an average of 4.2 days later [14], and this corresponds remarkably closely with the 63% lower risk between passive antibody therapies and vaccination observed in this study (Fig J in S1 Appendix). Therefore, neutralising antibodies present at the time of breakthrough infection (either due to vaccination or passive antibody administration) may be sufficient to provide the majority of protection against progression to severe outcomes. One way to further address this would be to consider the effects of prophylactic antibodies on progression to severe COVID-19 (in breakthrough infection). However, such studies are limited, with mixed results, providing no clarity on whether passively administered antibodies being present at the time of breakthrough infection do indeed provide greater protection against progression or not [47–51]. Identification of this ‘protection gap’ raises a number of important questions about the role of serological and cellular immunity in protection from progression to severe COVID-19 and further studies are needed to clarify the relative contribution of these immune responses.

This study has several limitations. Firstly, while the large number of included clinical studies is a strength of this work, there was also a large degree of between study variability, as evidenced by the level 3 I^2^ value of 71.8%. These differences may have arisen from known differences in study design as well as other potential unmeasured confounders (e.g., differences in hospitalisation decisions in different countries, and at different stages of the pandemic). Studies differed in the reporting of characteristics such as time since last vaccination, what clinical outcome was classified as severe, and what statistical risk measure was used to report vaccine protection (Table B in S1 Appendix). Furthermore, all studies were observational in nature and thus have an increased risk of unintended bias influencing outcomes compared to randomised controlled trials. In particular, these studies included only individuals with SARS-CoV-2 infection, thus eligibility for inclusion in the studies was not independent of vaccination status. There may also be other unmeasured differences between those vaccinated individuals who were infected and those who were not, limiting the strength of the evidence presented here for establishing a causal relationship [52]. Additionally, many included studies utilised databases that did not record symptoms at the time of a positive test. Consequently, the inclusion of people with asymptomatic infection likely varied across studies according to local testing practices, particularly whether testing was predominately carried out on symptomatic people or included routine surveillance testing. These differences in identifying cases will likely contribute to differences in the observed risk of progression, and thus protection estimates. We also cannot rule out the possibility of publication bias in the studies of vaccine protection against progression given some evidence of funnel plot asymmetry (Fig I in S1 Appendix), which may exaggerate the level of protection. Despite these potential sources of bias which cannot be excluded, the agreement of our analysis with the prediction derived in 2021 using data from randomised controlled trials (Fig 4), is somewhat encouraging.

As well as the potential for bias in the studies themselves (discussed above), our analysis is based on aggregate data rather than individual data, raising the possibility of ecological bias (i.e., the association between neutralising antibodies and vaccine effectiveness against progression may hold at the aggregate level but not at the individual level). The estimated protection from progression to severe COVID-19 achieved by passive antibody administration was also based on a meta-analysis of aggregate rather than individual data. Encouragingly, similar data aggregation approaches were successfully used to establish the first evidence of associations between neutralising antibody titres and vaccine efficacy against symptomatic and severe COVID-19 [3,10], and the associations in those studies have since been confirmed in randomised controlled trials at the individual level [6,7,11,53]. Future analysis of available individual-level data and comparison to the results seen here is likely to provide further valuable insights.

Importantly, neutralising antibody titres were not reported in the included clinical studies themselves but had to be predicted based on details of infecting variant, vaccine received, and time since last dose of the individuals included in the clinical studies. These were then matched to neutralising antibody data obtained from a separate meta-analysis of neutralising antibody titres from vaccinated populations (extracted from the Stanford database) [12]. The meta-analysis of neutralisation titres combines the reported GMTs across multiple assays and has the advantage of not being dependent on the results of a single assay. However, this approach also has a number of limitations, including that it does not explicitly account for differences in assays such as detection limits or other aspects of experimental design. Furthermore, the neutralisation titre estimates used in our analysis were from studies reporting titres from a general vaccinated population. However, the clinical progression studies include only the sub-population of vaccinated individuals who are already infected (who will more likely be those with lower titres) [3,6,7]. For example, the ratio in titres between cases and the general vaccinated population observed in RCTs for vaccines including Ad.COV2.S, ChAdOx2, Novavax and Moderna were 0.65, 0.45, 0.29, and 0.65, respectively [7,54–56]. Thus, the neutralising titres used here are likely an overestimate of the titres of those with breakthrough infection and so lower titres may be required for protection against progression than is indicated by our meta-analysis presented here. Finally, the matching process itself is likely imperfect, such that we cannot guarantee that the populations in the clinical studies themselves are well represented by the populations in the studies of neutralisation titres. Although, encouragingly in our analysis, the addition of our matched neutralising antibody titres was able to replace vaccine regimen and time since last dose as covariates in explaining differences in vaccine protection.

Despite the limitations of the data available and aggregated in this study, the association between neutralising antibody titres and protection against progression was robust to the inclusion of covariates, the use of death as a more objective clinical endpoint, the exclusion of studies with unknown or high rates of prior infection, the exclusion of studies with substantially higher rates of hospitalisation than others, as well as the removal of both influential observations and influential studies (Tables L-M and Figs F-H in S1 Appendix). While this work is a meta-analysis of the available evidence it should not be mistaken for providing a definitive assessment of evidence for clinical decisions. Instead, it is exploratory in nature and limited by the available data and the potential for risk of bias and confounding implicit in these observational studies, as well as the meta-analytic framework using different studies for clinical and immunological data (Table 1).

**Table 1:** Breakdown of included clinical and immunological studies. This table shows a comparison of the identified and included clinical and immunological studies. The number of observations shown here is the total number of estimates of vaccine effectiveness included in our analysis. Some studies contained multiple estimates of vaccine effectiveness, for example where results were reported separately for different vaccines, variants and/or different times since last vaccination.

	Clinical studies	Immunological studies
**Overall summary**		
#studies (# obs)		
Cohort	21 (118)	
Case-control	4 (19)	
Live virus		152 (927)
Pseudovirus		171 (1,229)
**Total observations** ^**#**^	**137**	**2,156**
**Total individuals**	**14,656,918**	**24,309**
**Observations by characteristic**		
**Variant**	**137**	**2,156**
WT	0	755
Pre-Omicron	68	765
Omicron	69	636
**Vaccine type**	**137**	**2,156**
mRNA	88	1,562
Inactivated	13	162
Vector	26	294
Inactivated (2 doses) + mRNA dose	2	45
Vector (2 doses) + mRNA dose	8	93
**Doses**	**137**	**2,156**
Primary course*	96	1,409
3 doses	40	696
4 doses	1	51

*Primary course is 1 dose for Ad26.COV2.S and 2 doses for all other vaccines

Neutralising antibodies have been used in immunobridging studies to support the licensure of both vaccines and monoclonal antibodies. However, despite the large number of studies examining the role of neutralising antibodies in protection from symptomatic COVID-19, their role in protection from severe infection remains less well understood [10,11,57]. Given that prevention of severe infection is a critical objective of COVID-19 interventions, a deeper understanding of the association between neutralising antibodies and severe disease is essential. In this study, we address this gap by providing direct evidence of the mechanistic relationship between neutralising antibody titres and protection against progression to severe COVID-19.

## Supporting information

S1 Appendix Supplementary material. (PDF)

## Abbreviations

AICc: Akaike information criterion
aRR: adjusted relative risk
aVE: adjusted vaccine effectiveness
GMT: geometric mean neutralisation titre
RR: relative risk.

## References

[1] Earle KA, Ambrosino DM, Fiore-Gartland A, Goldblatt D, Gilbert PB, Siber GR, et al. Evidence for antibody as a protective correlate for COVID-19 vaccines. Vaccine. 2021;39(32):4423–8. doi: 10.1016/j.vaccine.2021.05.063 34210573 PMC8142841

[2] Graña C, Ghosn L, Evrenoglou T, Jarde A, Minozzi S, Bergman H, et al. Efficacy and safety of COVID‐19 vaccines. Cochrane Database Syst Rev. 2022;(12). doi: 10.1002/14651858.CD015477 PMC9726273 36473651

[3] Khoury DS, Cromer D, Reynaldi A, Schlub TE, Wheatley AK, Juno JA, et al. Neutralizing antibody levels are highly predictive of immune protection from symptomatic SARS-CoV-2 infection. Nat Med. 2021;27(7):1205–11. doi: 10.1038/s41591-021-01377-8 34002089

[4] Kent SJ, Khoury DS, Reynaldi A, Juno JA, Wheatley AK, Stadler E. Disentangling the relative importance of T cell responses in COVID-19: leading actors or supporting cast? Nat Rev Immunol. 2022;22(6):387–97. doi: 10.1038/s41577-022-00716-1 35484322 PMC9047577

[5] Zonozi R, Walters LC, Shulkin A, Naranbhai V, Nithagon P, Sauvage G, et al. T cell responses to SARS-CoV-2 infection and vaccination are elevated in B cell deficiency and reduce risk of severe COVID-19. Sci Transl Med. 2023;15(724):eadh4529. doi: 10.1126/scitranslmed.adh4529 38019932 PMC12949954

[6] Feng S, Phillips DJ, White T, Sayal H, Aley PK, Bibi S, et al. Correlates of protection against symptomatic and asymptomatic SARS-CoV-2 infection. Nat Med. 2021;27(11):2032–40. doi: 10.1038/s41591-021-01540-1 34588689 PMC8604724

[7] Gilbert PB, Montefiori DC, McDermott AB, Fong Y, Benkeser D, Deng W. Immune correlates analysis of the mRNA-1273 COVID-19 vaccine efficacy clinical trial. Science. 2022;375(6576):43–50. doi: 10.1126/science.abm3425 34812653 PMC9017870

[8] Stadler E, Burgess MT, Schlub TE, Khan SR, Chai KL, McQuilten ZK, et al. Monoclonal antibody levels and protection from COVID-19. Nat Commun. 2023;14(1):4545. doi: 10.1038/s41467-023-40204-1 37507368 PMC10382502

[9] Follmann D, O’Brien MP, Fintzi J, Fay MP, Montefiori D, Mateja A, et al. Examining protective effects of SARS-CoV-2 neutralizing antibodies after vaccination or monoclonal antibody administration. Nat Commun. 2023;14(1):3605. doi: 10.1038/s41467-023-39292-w 37330602 PMC10276829

[10] Cromer D, Steain M, Reynaldi A, Schlub TE, Khan SR, Sasson SC, et al. Predicting vaccine effectiveness against severe COVID-19 over time and against variants: a meta-analysis. Nat Commun. 2023;14(1):1633. doi: 10.1038/s41467-023-37176-7 36964146 PMC10036966

[11] Carpp LN, Hyrien O, Fong Y, Benkeser D, Roels S, Stieh DJ, et al. Neutralizing antibody correlate of protection against severe-critical COVID-19 in the ENSEMBLE single-dose Ad26.COV2.S vaccine efficacy trial. Nat Commun. 2024;15(1):9785. doi: 10.1038/s41467-024-53727-y 39532861 PMC11557889

[12] Tzou PL, Tao K, Pond SLK, Shafer RW. Coronavirus Resistance Database (CoV-RDB): SARS-CoV-2 susceptibility to monoclonal antibodies, convalescent plasma, and plasma from vaccinated persons. PLoS One. 2022;17(3):e0261045. doi: 10.1371/journal.pone.0261045 35263335 PMC8906623

[13] Bates D, Mächler M, Bolker B, Walker S. Fitting linear mixed-effects models using lme4. J Stat Softw. 2015;67(1):1–48. doi: 10.18637/jss.v067.i01

[14] Stadler E, Chai KL, Schlub TE, Cromer D, Khan SR, Polizzotto MN, et al. Determinants of passive antibody efficacy in SARS-CoV-2 infection: a systematic review and meta-analysis. Lancet Microbe. 2023;4(11):e883–92. doi: 10.1016/S2666-5247(23)00194-5 37924835

[15] Cummings P. The relative merits of risk ratios and odds ratios. Arch Pediatr Adolesc Med. 2009;163(5):438–45. doi: 10.1001/archpediatrics.2009.31 19414690

[16] Viechtbauer W. Conducting Meta-Analyses in R with the metafor Package. J Stat Softw. 2010;36(3):1–48. doi: 10.18637/jss.v036.i03

[17] Bartoń K. MuMIn: Multi-Model Inference. 2025. doi: 10.32614/CRAN.package.MuMIn

[18] Butt AA, Yan P, Shaikh OS, Mayr FB, Omer SB. Rate and risk factors for severe/critical disease among fully vaccinated persons with breakthrough severe acute respiratory syndrome coronavirus 2 (SARS-CoV-2) infection in a high-risk national population. Clin Infect Dis. 2022;75(1):e849–56. doi: 10.1093/cid/ciab1023 34893812 PMC8689859

[19] Fournier PE, Houhamdi L, Colson P, Cortaredona S, Delorme L, Cassagne C. SARS-CoV-2 vaccination and protection against clinical disease: a retrospective study, Bouches-du-Rhône District, Southern France, 2021. Front Microbiol. 2021;12:796807. doi: 10.3389/fmicb.2021.796807 35116013 PMC8803903

[20] Hu Z, Tao B, Li Z, Song Y, Yi C, Li J, et al. Effectiveness of inactivated COVID-19 vaccines against severe illness in B.1.617.2 (Delta) variant-infected patients in Jiangsu, China. Int J Infect Dis. 2022;116:204–9. doi: 10.1016/j.ijid.2022.01.030 35065255 PMC8769614

[21] Ismail AlHosani F, Eduardo Stanciole A, Aden B, Timoshkin A, Najim O, Abbas Zaher W, et al. Impact of the Sinopharm’s BBIBP-CorV vaccine in preventing hospital admissions and death in infected vaccinees: results from a retrospective study in the emirate of Abu Dhabi, United Arab Emirates (UAE). Vaccine. 2022;40(13):2003–10. doi: 10.1016/j.vaccine.2022.02.039 35193793 PMC8857641

[22] Perrella A, Bisogno M, D’Argenzio A, Trama U, Coscioni E, Orlando V. Risk of SARS-CoV-2 infection breakthrough among the non-vaccinated and vaccinated population in Italy: a real-world evidence study based on big data. Healthcare (Basel). 2022;10(6):1085. doi: 10.3390/healthcare10061085 35742137 PMC9222607

[23] Smoll NR, Al Imam MH, Shulz C, Booy R, Khandaker G. The effectiveness of vaccination for preventing hospitalisation with COVID-19 in regional Queensland: a data linkage study. Med J Aust. 2023;219(4):162–5. doi: 10.5694/mja2.52019 37400415

[24] Tomioka K, Uno K, Yamada M. Association between vaccination status and COVID-19-related health outcomes among community-dwelling COVID-19 patients in Nara, Japan. Environ Health Prev Med. 2023;28:7. doi: 10.1265/ehpm.22-00199 36682815 PMC9884564

[25] Veneti L, Valcarcel Salamanca B, Seppälä E, Starrfelt J, Storm ML, Bragstad K, et al. No difference in risk of hospitalization between reported cases of the SARS-CoV-2 Delta variant and Alpha variant in Norway. Int J Infect Dis. 2022;115:178–84. doi: 10.1016/j.ijid.2021.12.321 34902584 PMC8664610

[26] Tomioka K, Uno K, Yamada M. Association between vaccination status and severe health consequences among community-dwelling COVID-19 patients during Omicron BA.1/BA.2 and BA.5-predominant periods in Japan. Environ Health Prev Med. 2023;28:35. doi: 10.1265/ehpm.23-00061 37286499 PMC10287986

[27] Albreiki M, Mousa M, Azman SK, Vurivi H, Alhalwachi Z, Alshehhi F, et al. Risk of hospitalization and vaccine effectiveness among COVID-19 patients in the UAE during the Delta and Omicron outbreaks. Front Immunol. 2023;14:1049393. doi: 10.3389/fimmu.2023.1049393 36860855 PMC9969353

[28] Bager P, Wohlfahrt J, Bhatt S, Stegger M, Legarth R, Møller CH, et al. Risk of hospitalisation associated with infection with SARS-CoV-2 omicron variant versus delta variant in Denmark: an observational cohort study. Lancet Infect Dis. 2022;22(7):967–76. doi: 10.1016/S1473-3099(22)00154-2 35468331 PMC9033212

[29] Bohnert AS, Kumbier K, Rowneki M, Gupta A, Bajema K, Hynes DM, et al. Adverse outcomes of SARS-CoV-2 infection with delta and omicron variants in vaccinated versus unvaccinated US veterans: retrospective cohort study. BMJ. 2023;381:e074521. doi: 10.1136/bmj-2022-074521 37220941

[30] Butt AA, Dargham SR, Chemaitelly H, Al Khal A, Tang P, Hasan MR, et al. Severity of illness in persons infected with the SARS-CoV-2 delta variant vs beta variant in Qatar. JAMA Intern Med. 2022;182(2):197–205. doi: 10.1001/jamainternmed.2021.7949 34935861 PMC8696690

[31] Davies M-A, Morden E, Rousseau P, Arendse J, Bam J-L, Boloko L, et al. Outcomes of laboratory-confirmed SARS-CoV-2 infection during resurgence driven by Omicron lineages BA.4 and BA.5 compared with previous waves in the Western Cape Province, South Africa. Int J Infect Dis. 2023;127:63–8. doi: 10.1016/j.ijid.2022.11.024 36436752 PMC9686046

[32] Greene SK, Levin-Rector A, Kyaw NTT, Luoma E, Amin H, McGibbon E, et al. Comparative hospitalization risk for SARS-CoV-2 omicron and delta variant infections, by variant predominance periods and patient-level sequencing results, New York City, August 2021-January 2022. Influenza Other Respir Viruses. 2023;17(1):e13062. doi: 10.1111/irv.13062 36317297 PMC9835408

[33] Hernández-Ávila M, Vieyra-Romero WI, Gutiérrez-Díaz HO, Zepeda-Tello R, Alpuche-Aranda C, Hernández-Ávila JE, et al. The omicron wave in Mexico: vaccine protection against progression to severe Covid-19 in SARS-CoV-2-infected workers. Salud Publica Mex. 2023;66(1, ene-feb):85–94. doi: 10.21149/15125 38065107

[34] Lewnard JA, Hong VX, Patel MM, Kahn R, Lipsitch M, Tartof SY. Clinical outcomes associated with SARS-CoV-2 Omicron (B.1.1.529) variant and BA.1/BA.1.1 or BA.2 subvariant infection in Southern California. Nat Med. 2022;28(9):1933–43. doi: 10.1038/s41591-022-01887-z 35675841 PMC10208005

[35] Mayr FB, Talisa VB, Castro AD, Shaikh OS, Omer SB, Butt AA. COVID-19 disease severity in US Veterans infected during Omicron and Delta variant predominant periods. Nat Commun. 2022;13(1):3647. doi: 10.1038/s41467-022-31402-4 35752687 PMC9233663

[36] Nyberg T, Ferguson NM, Nash SG, Webster HH, Flaxman S, Andrews N, et al. Comparative analysis of the risks of hospitalisation and death associated with SARS-CoV-2 omicron (B.1.1.529) and delta (B.1.617.2) variants in England: a cohort study. Lancet. 2022;399(10332):1303–12. doi: 10.1016/S0140-6736(22)00462-7 35305296 PMC8926413

[37] Tang F, Hammel IS, Andrew MK, Ruiz JG. COVID-19 mRNA vaccine effectiveness against hospitalisation and death in veterans according to frailty status during the SARS-CoV-2 delta (B.1.617.2) variant surge in the USA: a retrospective cohort study. Lancet Healthy Longev. 2022;3(9):e589–98. doi: 10.1016/S2666-7568(22)00166-0 PMC9342932 35935474

[38] Pool SNT, Shroff EH, Chetty A, Lewis L, Nonhlanhla YZ, Abdool Karim SS. Effectiveness of ChAdOx1 nCoV-19 and BBIBP-CorV vaccines against COVID-19-associated hospitalisation and death in the Seychelles infected adult population. PLoS One. 2024;19(4):e0299747. doi: 10.1371/journal.pone.0299747 38578809 PMC10997067

[39] Varea-Jiménez E, Aznar Cano E, Vega-Piris L, Martínez Sánchez EV, Mazagatos C, García San Miguel Rodríguez-Alarcón L, et al. Comparative severity of COVID-19 cases caused by Alpha, Delta or Omicron SARS-CoV-2 variants and its association with vaccination. Enferm Infecc Microbiol Clin (Engl Ed). 2024;42(4):187–94. doi: 10.1016/j.eimce.2022.11.021 36737369 PMC9890374

[40] Veneti L, Bøås H, Bråthen Kristoffersen A, Stålcrantz J, Bragstad K, Hungnes O, et al. Reduced risk of hospitalisation among reported COVID-19 cases infected with the SARS-CoV-2 Omicron BA.1 variant compared with the Delta variant, Norway, December 2021 to January 2022. Euro Surveill. 2022;27(4):2200077. doi: 10.2807/1560-7917.ES.2022.27.4.2200077 35086614 PMC8796289

[41] Wei Y, Jia KM, Zhao S, Hung CT, Mok CKP, Poon PKM, et al. Estimation of vaccine effectiveness of coronaVac and BNT162b2 against severe outcomes over time among patients with SARS-CoV-2 omicron. JAMA Netw Open. 2023;6(2):e2254777. doi: 10.1001/jamanetworkopen.2022.54777 36735253 PMC9898822

[42] Whitaker HJ, Tsang RSM, Byford R, Aspden C, Button E, Sebastian Pillai P, et al. COVID-19 vaccine effectiveness against hospitalisation and death of people in clinical risk groups during the Delta variant period: English primary care network cohort study. J Infect. 2023;87(4):315–27. doi: 10.1016/j.jinf.2023.08.005 37579793

[43] Khoury DS, Cromer D, Reynaldi A, Schlub TE, Wheatley AK, Juno JA, et al. What level of neutralising antibody protects from COVID-19? medRxiv. 2021. doi: 10.1101/2021.03.09.21252641

[44] Plotkin SA. Correlates of protection induced by vaccination. Clin Vaccine Immunol. 2010;17(7):1055–65. doi: 10.1128/CVI.00131-10 20463105 PMC2897268

[45] Casadevall A, Joyner MJ, Pirofski L-A, Senefeld JW, Shoham S, Sullivan D, et al. Convalescent plasma therapy in COVID-19: unravelling the data using the principles of antibody therapy. Expert Rev Respir Med. 2023;17(5):381–95. doi: 10.1080/17476348.2023.2208349 37129285

[46] Montgomery H, Hobbs FDR, Padilla F, Arbetter D, Templeton A, Seegobin S, et al. Efficacy and safety of intramuscular administration of tixagevimab-cilgavimab for early outpatient treatment of COVID-19 (TACKLE): a phase 3, randomised, double-blind, placebo-controlled trial. Lancet Respir Med. 2022;10(10):985–96. doi: 10.1016/S2213-2600(22)00180-1 35688164 PMC9173721

[47] Haidar G, Thomas S, Loubet P, Baker RI, Benfield T, Boonyaratanakornkit J, et al. Efficacy and safety of sipavibart for prevention of COVID-19 in individuals who are immunocompromised (SUPERNOVA): a randomised, controlled, double-blind, phase 3 trial. Lancet Infect Dis. 2025;25(7):813–26. doi: 10.1016/S1473-3099(24)00804-1 40015292

[48] Levin MJ, Ustianowski A, De Wit S, Launay O, Avila M, Templeton A, et al. Intramuscular AZD7442 (Tixagevimab-Cilgavimab) for prevention of Covid-19. N Engl J Med. 2022;386(23):2188–200. doi: 10.1056/NEJMoa2116620 35443106 PMC9069994

[49] Kertes J, Shapiro Ben David S, Engel-Zohar N, Rosen K, Hemo B, Kantor A, et al. Association between AZD7442 (Tixagevimab-Cilgavimab) administration and severe acute respiratory syndrome coronavirus 2 (SARS-CoV-2) infection, hospitalization, and mortality. Clin Infect Dis. 2023;76(3):e126–32. doi: 10.1093/cid/ciac625 35904210 PMC9384583

[50] Najjar-Debbiny R, Gronich N, Weber G, Stein N, Saliba W. Effectiveness of evusheld in immunocompromised patients: propensity score-matched analysis. Clin Infect Dis. 2023;76(6):1067–73. doi: 10.1093/cid/ciac855 36310534 PMC9623900

[51] Yan VKC, Yang Y, Wan EYF, Lai FTT, Chui CSL, Li X, et al. Real-world effectiveness and safety of tixagevimab-cilgavimab: a target trial emulation study. Drug Saf. 2024;47(10):1025–37. doi: 10.1007/s40264-024-01450-4 38916712 PMC11399184

[52] Hudgens MG, Halloran ME. Causal vaccine effects on binary postinfection outcomes. J Am Stat Assoc. 2006;101(473):51–64. doi: 10.1198/016214505000000970 19096723 PMC2603579

[53] Khoury DS, Schlub TE, Cromer D, Steain M, Fong Y, Gilbert PB, et al. Correlates of protection, thresholds of protection, and immunobridging among persons with SARS-CoV-2 infection. Emerg Infect Dis. 2023;29(2):381–8. doi: 10.3201/eid2902.221422 36692375 PMC9881762

[54] Benkeser D, Fong Y, Janes HE, Kelly EJ, Hirsch I, Sproule S, et al. Immune correlates analysis of a phase 3 trial of the AZD1222 (ChAdOx1 nCoV-19) vaccine. NPJ Vaccines. 2023;8(1):36. doi: 10.1038/s41541-023-00630-0 36899062 PMC10005913

[55] Fong Y, Huang Y, Benkeser D, Carpp LN, Áñez G, Woo W, et al. Immune correlates analysis of the PREVENT-19 COVID-19 vaccine efficacy clinical trial. Nat Commun. 2023;14(1):331. doi: 10.1038/s41467-022-35768-3 36658109 PMC9851580

[56] Fong Y, McDermott AB, Benkeser D, Roels S, Stieh DJ, Vandebosch A, et al. Immune correlates analysis of the ENSEMBLE single Ad26.COV2.S dose vaccine efficacy clinical trial. Nat Microbiol. 2022;7(12):1996–2010. doi: 10.1038/s41564-022-01262-1 36357712 PMC10166187

[57] Cromer D, Steain M, Reynaldi A, Schlub TE, Wheatley AK, Juno JA, et al. Neutralising antibody titres as predictors of protection against SARS-CoV-2 variants and the impact of boosting: a meta-analysis. Lancet Microbe. 2022;3(1):e52–61. doi: 10.1016/S2666-5247(21)00267-6 34806056 PMC8592563

