# Supplementary material for: Neutralising antibodies and protection from progression to severe COVID-19: A meta-analysis
Source: PLoS Med. 2026 Sep 2;23(9):e1005230. doi: 10.1371/journal.pmed.1005230 (PMC13537689; doi:10.1371/journal.pmed.1005230)
Supplement: Supplementary file 1 [file pmed.1005230.s001.pdf]

Supplement

Supplementary materials 3

Search strategy – Clinical studies 3

Data extraction – Clinical studies 4

Vaccine protection against progression 5

Neutralising antibodies – Immunological studies 6

Linking neutralising antibody levels and disease progression data 7

Modelling vaccine protection against progression 7

Model including age group and variant as covariates 8

Sensitivity analyses 9

Comparison to prior work 10

Tables 12

Table A Summary of included clinical studies 12

Table B Characteristics of included clinical studies 16

Table C Vaccines not used for matching 18

Table D Vaccine regimen matches 18

Table E Variant matches 19

Table F Time since last dose matches 19

Table G Intercept only model of vaccine protection from progression 20

Table H Vaccine protection from progression model comparison 20

Table I Multivariate model of vaccine protection from progression 21

Table J Model with neutralising antibody titres as the only covariate 22

Table K Model comparison: neutralising antibodies plus covariates 22

Table L Models including age group and variant as covariates 23

Table M Sensitivity analyses 24

Table N Variant investigations 25

Figures 26

Figure A PRISMA flow diagram of included studies 26

Figure B Forest plots for pre-Omicron variants 27

Figure C Forest plots for Omicron variants 29

Figure D Vaccine protection against progression by age group 31

Figure E Neutralisation titres by vaccine regimen and variant 32

Figure F Alternate models 33

Figure G Influential observations 34

Figure H Leave-one-study-out analysis 35

Figure I Funnel plot 36
Figure J Effect of timing difference between antibody treatment and vaccination 37
References **38**

Supplementary materials

Search strategy – Clinical studies

A systematic search of the literature was performed using PubMed from December 01, 2020, to January 23rd, 2025. The PICOS framework (Population, Intervention, Control, Outcomes and Study type) was adapted to define the inclusion and exclusion criteria. Search terms were:

((((“COVID-19”) AND

(“vaccin*”) AND

((“risk*” OR “odds ratio”) AND

(“progress*” OR “hospital*”)) AND

(“variant” OR "COVID-19 infected"))) NOT

(“animals” NOT “humans”)) NOT

(“review” OR “systematic”)

Articles were screened by two authors (KME and AM) according to the following criteria:

Inclusion criteria

Studies which assessed outcomes in a population who were already infected with SARS-CoV-2 and where rates of progression to severe disease were compared between vaccinated and unvaccinated people, regardless of prior infection rates in the cohort.

Exclusion criteria

- Animal or in-vitro studies
- Full text not in English
- Reviews, meta-analyses, case reports
- Studies assessing the effectiveness of COVID-19 treatments
- Studies that restricted enrolment to only paediatric or only elderly people
- Studies exclusively of residents of long-term care facilities
- Studies solely in people with other medical conditions e.g. kidney failure, solid organ transplant, undergoing cancer treatments, multiple sclerosis, pregnancy
- Studies that recruited from ED presentations or hospital admissions
- Studies that did not compare outcomes in vaccinated vs unvaccinated populations (e.g. only compared boosted to primary course, or compared one vaccine to another)
- Studies that considered outcomes in the overall population rather than in those known to be infected
- Studies that reported outcomes that combined pre-Omicron and Omicron infections

- Studies that reported outcomes that combined doses received (e.g. outcome reported for a combination of complete primary course and boosted vaccinees)
- Studies that did not provide information about which vaccines had been used
- Studies where the results were reported for a group who received multiple vaccines, and the most common vaccine was received by less than 50% of the group

Due to limited vaccine supply and preferential vaccine rollout to higher risk populations, non-randomised studies had the potential to be significantly confounded by large differences in the underlying risk of severe outcomes between the vaccinated and unvaccinated cohorts. Given this, we excluded any non-randomised study that made no attempt to account for this potential confounding (e.g. where only raw count data was reported). The minimum requirement for inclusion was that a study adjusted for age, matched on age, or stratified by age. The systematic review was not pre-registered.

Data extraction – Clinical studies

We extracted all data from the included studies on administered vaccines (including type, number of doses, and timing of last dose where available), SARS-CoV-2 variant (typed at infection or reported to be the dominant circulating variant at the time of the study), and reported results relating to vaccine protection from disease progression. This included details on both the outcomes used to define progression (hospital admission, ICU admission, death, or composites of these outcomes), and statistical risk measure used to compare the outcomes between the vaccinated and unvaccinated groups (i.e. odds ratio (OR), relative risk (RR), hazard ratio (HR), incidence proportion (IP), cumulative incidence ratio (CIR) and whether they were adjusted measures). We also extracted other descriptive data where available, such as study design, enrolment dates, mean/median age of participants, rates of prior infection, and number of participants. All data was independently extracted by two authors (KME and AM), with any discrepancies resolved through discussion. Where required, data was extracted from figures using WebPlotDigitizer (version 4.7). If two studies reported data from the same or an overlapping cohort of individuals, only the publication containing the most complete and disaggregated data was used.

For two studies [1, 2] it was not clearly stated what the infecting variants were, in both cases we have classified these as being a combination of Alpha and Delta variants based on the enrolment dates of the studies. For studies where the infecting variant was described only as Omicron without including subvariant information, we used enrolment dates of the study and classified subvariants using the following dates:

- From 01 Dec 2021 to 31 Jan 2022 – BA.1 only
- From 01 Feb 2022 to 30 Jun 2022 – BA.1 and BA.2
- From 30 Jun 2022 to 30 Sep 2022 – BA.5

These dates were based on CDC data on the prevalence of circulating variants [3]. Studies that calculated a single measure of vaccine effectiveness for groups containing both pre-Omicron and Omicron variants were excluded.

When extracting age data from studies, the mean or median was used where this was reported. Where mean or median were not available, and age in the underlying studies was reported as a number (%) in various age groups, the midpoint of the age range containing the median was used. One study[4] reported age as an overall mean and as a number (%) by age groups, and reported vaccine protection against progression stratified by age, so we extracted ages based on the number (%) reported for each stratum instead of the overall mean.

One paper which described their included population as "COVID-19 cases" was excluded as we determined that the case definition used in this study was not consistent with other included studies [5]. The description of the included cases showed that 75% were asymptomatic, and with approximately half of the individuals having Ct>40 (N gene Ct>40 in 42% of cases and Ct 35-40 in 21%, ORF 1 gene Ct>40 in 51% of cases and Ct 35-40 in 21%).

Vaccine protection against progression

When investigating what factors were predictive of vaccine protection from progression, the following potential covariates were considered:

- Vaccine regimen (defined as the combination of vaccine type and number of dose)
- Variant (categorised as either pre-Omicron or Omicron)
- Age (mean or median for the cohort), categorised as <40 or ≥ 40 years
- Time since last dose (days) (detailed below)
- Study type (cohort vs case-control)
- Statistical risk measure (aOR, aHR, aRR, etc.)
- The study outcome that was used from each study in the analysis (i.e. hospitalisation for 20/25 studies, and ICU for 5/25).

Time since last vaccine dose was not reported at all in 15 (60%) studies, covering 36 (26%) of all observations. Where it was reported, time since last dose was reported as an aggregate measure (mean or median) in 7 (28%) studies, containing 23 (17%) observations, and with

results stratified by time since last dose in 8 (32%) studies, containing 78 (57%) observations (Supp. Table B). When including time since last dose in our analysis we used the following values:

i. Where stratified results were reported we used the midpoint of the relevant time strata;
ii. Where an aggregate measure was reported this was used (mean/median); or
iii. Where no time since last dose was available, we used mean imputation, using the average of the observations from (ii)

Neutralising antibodies – Immunological studies

The Stanford University Coronavirus antiviral and resistance database was searched for all vaccinee plasma susceptibility data (database accessed on 12th Oct 2023) [6]. We excluded results that:

- Only reported fold-drop between variants rather than actual titres for each variant
- Were surrogate neutralisation assays rather than live virus or pseudo virus assays
- Were in animal models
- Were against viral lines listed as having specific mutations but otherwise not classified as a particular SARS-CoV-2 variant
- Were in people with known prior infection

We included results for both the baseline and comparator variants, provided they were either for the ancestral strain, or a variant present in the clinical studies. Time since last dose corresponding to each neutralisation titre was reported as a categorical variable with three categories: <1 month, 2-6 months and greater than 6 months.

Neutralising antibody data were aggregated with a linear mixed-effects model (using the lmer function from the lme4 package in R) with fixed effects for regimen, time since last dose, variant and assay type (i.e. live vs psuedovirus neutralisation), and random intercepts for study. We then considered the inclusion of an interaction term between regimen and time since last dose (to allow antibody waning to differ between vaccines), and an interaction term between variant and time since last dose (to allow antibody waning to differ between variants).

The Stanford database contained many more results for mRNA vaccines than other vaccine types and contained more data at <1-month post-vaccination compared to 2-6 months and >6 months post-vaccination. Decline in antibodies with time did not differ between variants (p =

0.147 by LRT, for interaction between time and variant) but did differ between vaccine regimens ($p<0.0001$ by LRT, for interaction between time and vaccine regimen). Our final model included fixed effects for regimen, time since last dose, variant, assay type, and an interaction term between regimen and time since last dose, as well as a random intercept for study. The geometric mean titres estimated by our meta-regression were markedly lower for Omicron variants than pre-Omicron variants and declined with time since vaccination. Live virus assays consistently reported lower titres than pseudovirus assays (Supp. Figure E).

Linking neutralising antibody levels and disease progression data

Neutralisation data were matched to progression data based on regimen, time since last dose, and variant, with live virus assay neutralisation results being used for all matches. In cases where there was a "many-to-one" mapping of regimen, e.g., a progression result for combined mRNA being matched to neutralisation titres for both BNT162b2 and mRNA-1273, an inverse variance weighted average estimate of the titres for all matching regimens was used (Supp. Table D). In cases where the reported progression result was for multiple variants, the (non-weighted) geometric mean of the relevant titre estimates was used (Supp. Table E). For time since last dose, clinical results with no indication of time since last dose were matched to the geometric mean of titres for all three available timepoints from the neutralisation data, and in cases where the reported range of times from the progression result covered more than one of the available timepoints, the geometric mean of titres for all relevant timepoints was used (Supp. Table F). For cases where we needed to aggregate across more than one domain (e.g. multiple timepoints and multiple variants), the (unweighted) geometric mean across domains was used. For example, if we were matching 3 doses of any mRNA vaccine and Omicron BA.1/BA.2, we first calculated the weighted geometric mean for 3 doses of any mRNA for both Omicron BA.1 and Omicron BA.2, then calculated the geometric mean of these two results to obtain our titre. There was one included paper [7] with progression data for a combination of Omicron BA.4 and Omicron BA.5, however there were only four results in the Stanford database for neutralisation titres against BA.4, so we matched this paper to BA.5 titres only. Full details of matches made are provided in Supp. Tables D - F.

Modelling vaccine protection against progression

To aggregate the estimates of protection from progression, we used a multilevel linear mixed effects meta-regression model. For the random effects structure, we found that the multilevel model with random intercepts at the study level (Level 3) and at the effect-size-within-study level (nested at Level 2), was a better fit to the data than a simpler model with between study

clustering only ($p < 0.001$ by LRT, estimated using REML). The estimated variance components/intraclass correlation coefficients (ICCs) (Supp. Tables H-L), indicate heterogeneity is predominately between studies (Level 3), with additional non-trivial within-study heterogeneity (Level 2), and minimal residual (Level 1) variance. This supported the choice to use a three-level nested structure.

Model including age group and variant as covariates

In our meta-regression of protection against progression from SARS-CoV-2 infection to severe COVID-19, we found that age group (i.e. categories, <40 and >40 years of age) and variant (pre-Omicron vs Omicron) remained significant covariates in our model, with lower vaccine effectiveness seen for older cohorts (Supp. Figure F(B)) and for Omicron variants (Supp. Figure F(A)).

Although we observed lower vaccine effectiveness in studies with older cohorts, after adjusting for age, we still observed vaccine protection from progression increase with neutralisation titres (Supp. Figure F(B), Supp. Table L). When matching disease progression data to neutralisation titres, we were unable to match neutralisation titres by age as this was not available. Given the existing evidence of lower immune responses to vaccination in older people [8], it is likely that the significant impact of age on vaccine effectiveness is at least partially due to older individuals having achieved lower than the population average levels of neutralisation for a given vaccine regimen and thus our estimates of the neutralising antibody titres are not representative for this group. We are unable to determine if it is the impact of age on titres, or a true difference in the association between titres and vaccine protection from progression by age, or a combination of both that means protection is lower in the older age groups, given the available data.

The reason for the difference in vaccine protection between pre-Omicron and Omicron variants, even after accounting for differences in neutralisation (since we matched antibody titres by variant and thus our analysis should have taken differences in antibody titres by variant into account), is less clear. To further investigate possible causes of this result, we performed exploratory analyses. Firstly, we considered only data in the neutralisation titre range where Omicron and pre-Omicron were overlapping (Supp. Table N) and found that variant was still a significant factor. We then limited our analysis to only those studies which contained both pre-Omicron and Omicron results, to reduce potential bias from unmeasured confounding between studies, and found that variant remained significant (Supp. Table N). Given the possibility that

estimates of vaccine protection against progression were impacted by changes in public health policy and social practises between the pre-Omicron and Omicron era, we then limited even further to only include studies that contained concurrent Delta and Omicron infections, where variant again remained significant (Supp. Table N). It is possible that the differences in vaccine protection from progression may be due to a fundamentally different risk of severe outcomes between pre-Omicron and Omicron variants [9]. Alternatively, it may be due to in vitro measurement effects such as higher uncertainty and a greater likelihood of limit of detection issues affecting the reported GMTs for Omicron vs pre-Omicron variants. In addition, changes in case ascertainment in the Omicron era may have impacted clinical studies. Our ability to investigate possible causes of this difference was limited due to the majority of the range of post-vaccination GMTs being non-overlapping between Omicron and pre-Omicron variants.

Sensitivity analyses

To assess the robustness of our observation that neutralising antibody titres are predictive of protection against progression to severe outcomes, we performed additional sensitivity analyses. First, since the clinical studies were all observational, the clinicians would not have been blinded to individuals' vaccination history in most cases. This has the potential to cause a bias in decisions to hospitalise, due to knowledge of individuals' vaccination status, or due to differing guidelines for hospitalisation across time and between countries. To avoid this potential for bias, we undertook a secondary analysis using only death as an endpoint for disease progression (in studies where this was available, 9/25 studies, totalling 76 observations), as this is less likely to be affected by the lack of blinding between practitioners and vaccine status, or regional treatment policies that used vaccination status in the decision to hospitalise or treat. We found neutralising antibodies were also significantly associated with protection against progression to death (RR 0.47 per $\log_{10}$ increase in GMT, 95% CI [0.40, 0.55], $p < 0.001$) (Supp. Table M).

The assumption of the equivalence of OR and RR is only valid when events are rare [10], so progression rates >10% may be problematic for this assumption. We noted that four of our cohort studies had overall rates of hospitalisation >10% (three of which were studies in older cohorts of US veterans who were expected to be at higher risk of severe outcomes). We undertook a secondary analysis excluding these four studies and found that neutralising antibodies were still significantly associated with protection against progression to severe outcomes (RR 0.55 per $\log_{10}$ increase in GMT, 95% CI [0.49, 0.62], $p < 0.001$) (Supp. Table M).

We next considered whether any individual observations or studies were unduly influencing the results of our regression analysis. We reran our analysis excluding any observations identified as strongly influential (Cook's distance, cutoff = 0.25) and found no change to our conclusions (Supp. Figure G) indicating that individual observations are not influencing our results. We also performed a leave-one-out analysis on the studies to assess the importance of any one study on the identified association and found that the estimated model parameters (intercept range: -0.438 to -0.335 and slope range: -0.591 to -0.498) agree with the 95% CI of the fit to all the data (intercept -0.693 to -0.100, slope -0.672 to -0.452) (Supp. Figure H).

Comparison to prior work

To compare the relationship between neutralisation titres and protection against progression to severe outcomes observed in this study with that predicted by Khoury et al.[11], we used the protection curves against symptomatic infection and severe infection from Khoury et al. (reproduced here in Figure 1A and 1B) and derived the protection against progression from symptomatic to severe by first computing the relative risk as 1-effectiveness (Figure 4A). At any given neutralisation titre, the RR against progression is given by:

$$RR_{progression} = \frac{RR_{severe}}{RR_{symptomatic}}.$$

To obtain confidence bands for the estimated relative risk of progression we performed parametric bootstrapping. Specifically, we used the parameter estimates and covariance matrix of the simultaneous model fit for symptomatic and severe from Khoury et al. [11]. Using these we constructed a multivariate normal distribution, which was sampled 10,000 times (using the rmvnorm function from the mvtnorm package in R). We then calculated the relative risk of progression from these 10,000 sampled values at each level of fold-convalescent titre and used percentiles of these results to estimate the lower and upper bounds of the 95% confidence bands (red shaded region in Figure 4a).

We also compared the relationship between neutralisation titres and protection against progression to severe outcomes (observed in this study), with the dose-response relationship of passive antibody treatments and their effectiveness at preventing progression to severe outcomes (previously determined by Stadler et al. [12]). Restricting our analysis to neutralising antibody levels in the range of 0.1 to 10 fold-convalescent (the range that was common to both vaccination and antibody therapy), we quantified the observed "protection gap" between the effectiveness of vaccination and antibody treatment, by converting both curves to RR curves

using the formula $RR = 1 - Eff$. We then calculated the protection gap at any given neutralisation titre in the following way:

$$RR_{vaccine} = RR_{mAb} \times RR_{gap}$$

To obtain confidence bands for the protection gap we used parametric bootstrapping. Specifically, we used the parameter estimates and covariance matrices of the fitted models for both vaccination and monoclonal antibody treatment to describe multivariate normal distributions which were sampled 10,000 times (using the rmvnorm function from the mvtnorm package in R). We then calculated the gap in relative risk using these 10,000 sampled values at each level of fold-convalescent titre and used percentiles of these values to estimate the lower and upper bounds of the 95% confidence bands.

The average of the protection gap between vaccines and antibody administration across the range of neutralisation titres was calculated as the integral over the range divided by the length of the range.

Tables

Table A Summary of included clinical studies

Author	Country	Study design	Included ages	Included vaccines	Included variants	Enrolment dates	Number of participants	Clinical endpoint used in main analysis	Data source for risk	Risk measure reported
Al Hosani et al.[13]	UAE	Cohort	15+	BBIBP-CorV	Alpha, Beta	01 Sep 2020 to 01 May 2021	154,872	COVID-19 related hospital admission	Table 3	aHR
Albreiki et al.[4]	UAE	Cohort	All	BBIBP-CorV, BNT162b2	Delta, Omicron BA.1	28 May 2021 to 13 Jan 2022	4615	COVID-19 related hospital admission	Table 3	aOR
Bager et al.[14]	Denmark	Cohort	All	BNT162b2, mRNA-1273	Delta, Omicron BA.1	21 Nov 2021 to 19 Dec 2021	188,980	Hospital admission	Table 3, text of results[a]	aRR
Bohnert et al.[15]	USA	Cohort	18+ (veterans)	BNT162b2, mRNA-1273, Ad26.COV2.S	Delta, Omicron BA.1 and BA.2	01 Jul 2021 to 30 Jun 2022	279,989	Hospital admission	Tables 4-5	aOR
Butt et al. (a)[1]	USA	Case-control	18+ (veterans)	BNT162b2, mRNA-1273	Alpha, Delta	15 Dec 2020 to 30 Jun 2021	3456	Composite of ICU, MV and death	Table 3	aHR
Butt et al. (b)[16]	Qatar	Cohort	All	BNT162b2, mRNA-1273	Beta, Delta	22 Mar 2021 to 07 Jul 2021	902	Composite of COVID-19 related hospital admission, supp. O_2, MV or death	Table 4	aOR
Davies et al.[7]	South Africa	Cohort	20+	BNT162b2, Ad26.COV2.S	Omicron BA.4/BA.5[b]	01 May 2022 to 21 May 2022	3793	Composite of ICU, MV or steroid prescription	Text of results section	aHR

Fournier et al.[17]	France	Cohort	All	BNT162b2, mRNA-1273, Ad26.COV2.S, AZD1222	Alpha, Delta	18 Jan 2021 to 13 Aug 2021	10,566	Hospital admission	Table 3	aOR
Greene et al.[18]	USA	Cohort	All	BNT162b2, mRNA-1273	Delta, Omicron BA.1	01 Aug 2021 to 31 Jan 2022	646,852	Hospital admission	Table S2	aRR
Hu et al.[19]	China	Cohort	18+	CoronaVac, BBIBP-CorV	Delta	01 Jul 2021 to 31 Aug 2021	476	Severe illness[c]	Table 2	aOR
Lewnard et al.[20]	USA	Cohort	All	BNT162b2, mRNA-1273, Ad26.COV2.S	Delta, Omicron BA.1	15 Dec 2021 to 17 Jan 2022[d]	245,993	Symptomatic hospital admission	Table S13	aHR
Mayr et al.[21]	USA	Cohort	18+ (veterans)	BNT162b2, mRNA-1273	Delta, Omicron BA.1	01 Oct 2021 to 15 Jan 2022	45,682	Composite of hospital admission, ICU admission and death	Text of additional analyses section	aOR
Nyberg et al.[9]	England	Cohort	All	BNT162b2, mRNA-1273, AZD1222	Delta, Omicron BA.1	29 Nov 2021 to 09 Jan 2022	1,516,702	Hospital admission	Table S3	aHR
Perella et al.[2]	Italy	Cohort	18+	BNT162b2, mRNA-1273, AZD1222, Ad26.COV2.S	Alpha, Delta	08 Mar 2021 to 31 Oct 2021	171,921	ICU admission due to COVID-19	Table 2	aOR
Smoll et al.[22]	Australia	Cohort	18+	BNT162b2, mRNA-1273, AZD1222	Omicron BA.1 and BA.2	01 Jan 2022 to 31 Mar 2022	9,682	COVID-19 related hospital admission	Text of abstract	aRR
Tang et al.[23]	USA	Cohort	19+	BNT162b2, mRNA-1273	Delta	25 Jul 2021 to 30 Sep 2021	57,784	COVID-19 related hospital admission	Tables S5-S6	aOR
Tomioka et al. (a)[24]	Japan	Cohort	12+	BNT162b2, mRNA-1273	Delta[e]	01 Apr 2021 to 31 Mar 2022	3,121	Composite of ICU admission or COVID-19 related death	Additional file 7A	aIP[f]

Tomioka et al. (b)[25]	Japan	Cohort	12+	BNT162b2, mRNA-1273	Omicron BA.1, BA.2 and BA.5	01 Jan 2022 to 25 Sep 2022	69,827	Composite of COVID-19 related hospital admission or death	Figure 2	aCIR[g]
Varea-Jiminez et al.[26]	Spain	Case-Control	12+	BNT162b2, mRNA-1273, AZD1222, Ad26.COV2.S	Alpha, Delta, Omicron BA.1	01 Jun 2021 to 31 Jan 2022	33,169	COVID-19 related hospital admission	Tables 2 and S3	aOR
Veneti et al. (a)[27]	Norway	Cohort	All	BNT162b2, mRNA-1273, AZD1222, Ad26.COV2.S	Alpha, Delta	03 May 2021to 15 Aug 2021	20,055	COVID-19 related hospital admission	Table 2	aRR
Veneti et al. (b)[28]	Norway	Cohort	All	BNT162b2, mRNA-1273, AZD1222, Ad26.COV2.S	Delta, Omicron BA.1	06 Dec 2021 to 09 Jan 2022	91,005	COVID-19 related hospital admission	Table 3	aHR
Wei et al.[29]	Hong Kong	Case Control	18+	BNT162b2, CoronaVac	Omicron BA.1 and BA.2	01 Jan 2022 to 05 Jun 2022[h]	164,160	Composite of hospital admission or death	Table 2	aOR
Hernandez-Avila et al.[30]	Mexico	Cohort	16+	AZD1222, BNT162b2, mRNA-1273, CoronaVac, Ad26.COV2.S, Sputnik V, Ad5-nCoV[j]	Omicron BA.1 and BA.2	02 Jan 2022 to 05 Mar 2022	347,899	Hospital admission	Table 2	aRR
Pool et al.[31]	Seychelles	Cohort	18+	AZD1222, BBIBP-CorV	Beta, Delta	25 Jan 2021 to 30 Jun 2021	10,205	Composite of hospital admission or death	Table 2	aRR
Whitaker et al.[32]	England	Cohort	16+	AZD1222, BNT162b2	Delta	17 May 2021 to 06 Dec 2021	10,575,212	Hospital admission with at least 2-night stay	Table 2	aVE

[a] Text of results gives aRR split by time since last dose for individuals with Delta infections who received 3 doses. Omicron aRR of hospitalisation was derived from data in Table 3 which showed all results relative to reference of unvaccinated Delta infection.
[b] While this study included more variants and time periods, only the Omicron BA.4/BA.5 result was used, as that was the only one disaggregated by vaccine type, number of doses and variant (as needed for our analysis).
[c] Severe illness was defined as meeting at least one of the following criteria: (1) respiratory rate ≥30 breaths/min; (2) oxygen saturation measured by finger pulse oximeter during air inhalation ≤93% while at rest; (3) arterial partial pressure of oxygen (PaO2)/oxygen uptake concentration (FiO2) ≤300 mm Hg; and (4) aggravated clinical symptoms and pulmonary imaging showing that the lesion progressed more than 50% within 24-48 hours.
[d] There was a separate cohort enrolled later (Feb-Mar 2022) in the same paper which was not used as it did not have the data needed for our analysis.
[e] While this study included results for Omicron infections, these were not used here as they were a subset of the individuals included in Tomioka (b).
[f] aIP is adjusted incidence proportion.
[g] aCIR is adjusted cumulative incidence ratio.
[h] Patients who were hospitalised prior to week 6 of 2022 were excluded from the analysis as prior to this time all cases were hospitalised regardless of clinical status.
[j] This study reported results for Ad5-nCoV (CanSino) which were not included in our analysis since there were no neutralising antibody titres available for this clinical result to be matched to.
MV – mechanical ventilation

Table B Characteristics of included clinical studies

Characteristic		Studies n (%)	Observations‡ n (%)
	total	25 (100%)	137 (100%)
Study type			
	cohort	21 (84%)	118 (86%)
	case control	4 (16%)	19 (14%)
Prior infection rates			
	0%	5 (20%)	14 (10%)
	<10%	7 (28%)	70 (51%)
	10-20%	1 (4%)	3 (2%)
	not reported	12 (48%)	50 (36%)
Time since last dose§*			
	aggregate <3mths	3 (12%)	4 (3%)
	aggregate 3-6mths	6 (24%)	15 (11%)
	aggregate >6mths	2 (8%)	4 (3%)
	stratified <3mths	6 (24%)	38 (28%)
	stratified 3-6mths	7 (28%)	32 (23%)
	stratified >6mths	4 (16%)	8 (6%)
	not reported	15 (60%)	36 (26%)
Age – type¶			
	n (%) in age brackets	10 (40%)	74 (54%)
	median	10 (40%)	37 (27%)
	mean	5 (20%)	26 (19%)
Age			
	<40 yrs	16 (64%)	86 (63%)
	40-79 yrs	12 (48%)	51 (37%)
Least severe available endpoint			
	hospital admission	20 (80%)	130 (95%)
	ICU admission/mechanical ventilation	5 (20%)	7 (5%)
Reporting of least severe endpoint			
	all cause	12 (48%)	93 (68%)
	COVID-19 related	13 (52%)	44 (32%)
Death endpoint			
	reported	9 (36%)	76 (55%)
	not reported	16 (64%)	61 (45%)
Reporting of death outcome			
	all-cause death	7 (78%)	71 (93%)
	COVID-19 listed as cause	1 (11%)	1 (1%)
	not attributable to external cause	1 (11%)	4 (5%)
Statistical risk measure			
	odds ratio (OR)	9 (36%)	29 (21%)
	hazard ratio (HR)	6 (24%)	61 (45%)
	relative risk (RR)	5 (20%)	18 (13%)
	vaccine effectiveness (VE)	3 (12%)	23 (17%)
	incidence proportion (IP)	1 (4%)	1 (1%)
	cumulative incidence ratio (CIR)	1 (4%)	5 (4%)
Overall hospitalisation rates#			
	<5%	14 (56%)	92 (67%)

5-10%	3 (12%)	6 (4%)
>10%	4 (16%)	20 (15%)
N/A[#]	4 (16%)	19 (14%)
% receiving most common vaccine in a group[*]		
100%	17 (68%)	114 (83%)
95% to <100%	3 (12%)	13 (9%)
69 to 86.5%	6 (24%)	10 (7%)
Variant[*]		
Alpha	1 (4%)	1 (1%)
Alpha/Beta	1 (4%)	1 (1%)
Alpha/Delta	4 (16%)	4 (3%)
Beta/Delta	2 (8%)	4 (3%)
Delta	13 (52%)	58 (42%)
Omicron BA.1	8 (32%)	39 (28%)
Omicron BA.1/BA.2	5 (20%)	24 (18%)
Omicron BA.4/BA.5	1 (4%)	3 (2%)
Omicron BA.5	1 (4%)	3 (2%)

[ǂ]Observations refers to individual estimates of vaccine effectiveness included in our analysis. Some studies contained multiple estimates of vaccine effectiveness, for example where results were reported separately for different vaccines, variants and/or different times since last vaccination.

[§]Aggregate time since last dose (TSLD) categories are those where one overall measure (e.g. mean or median) was reported for a group of participants, while stratified are cases where groups were stratified by time since last dose and a separate estimate was provided for each stratum.

[*]TSLD/Variant/% receiving most common vaccine: study count >100% as some studies reported multiple results that fit into different categories.

[¶]Age type describes the way age was reported in the studies, either mean, median, or number and percentage of participants in given age brackets. Where numbers per age bracket were reported, the value used for age was the midpoint of the age bracket containing the median.

[#]Overall hospitalisation rates not known for case-control studies.

Table C Vaccines not used for matching

In some studies, not all vaccines received by the cohort were used to match to neutralisation titres. These were all studies where vaccine effectiveness was reported for a cohort in which the majority of individuals received mRNA vaccines, which is what the matched neutralisation titres were based on.

Study	Proportion who received mRNA vaccines	Minority vaccines received
Fournier et al.	72%	AZD1222, Ad26.COV2.S
Perella et al.	80%	AZD1222, Ad26.COV2.S
Smoll et al.	86.5%	AZD1222
Varea-Jiminez et al.	69-81%	AZD1222, Ad26.COV2.S
Veneti et al. (a)	83%	AZD1222, Ad26.COV2.S
Veneti et al. (b)	96-98%	AZD1222, Ad26.COV2.S

Table D Vaccine regimen matches

Vaccine regimen (Clinical studies)	Vaccine regimen (Neutralisation database)
1 dose Ad26.COV2.S	1 dose Ad26.COV2.S
2 doses AZD1222	2 doses AZD1222
2 doses BBIBP-CorV	2 doses BBIBP-CorV
2 doses BNT162b2	2 doses BNT162b2
2 doses CoronaVac	2 doses CoronaVac
2 doses Sputnik V	2 doses Sputnik V
2 doses CoronaVac or BBIBP-CorV	2 doses CoronaVac<br>2 doses BBIBP-CorV
2 doses mRNA-1273	2 doses mRNA-1273
2 doses any mRNA	2 doses BNT162b2<br>2 doses mRNA-1273<br>2 doses mRNA
2 doses AZ1222, with any mRNA third dose	2 doses AZD1222+ 1 dose BNT162b2<br>2 doses AZD1222+ 1 dose mRNA-1273<br>2 doses AZD1222+ 1 dose mRNA
CoronaVac primary with BNT162b2 third dose	2 doses CoronaVac + 1 dose BNT162b2
3 doses BNT162b2	3 doses BNT162b2
3 doses CoronaVac	3 doses CoronaVac
3 doses any mRNA	3 doses BNT162b2<br>3 doses mRNA-1273<br>3 doses mRNA
4 doses any mRNA	4 doses BNT162b2<br>4 doses mRNA-1273<br>4 doses mRNA

Matches made for vaccine regimen between progression studies and neutralisation titres. Note that for any row in right column with multiple entries, these were combined as the inverse variance weighted geometric mean of the titres for each entry.

Table E Variant matches

Variant (Clinical studies)	Variant (Neutralisation database)
Alpha	Alpha
Alpha/Beta	Alpha<br>Beta
Alpha/Delta	Alpha<br>Delta
Beta/Delta	Beta<br>Delta
Delta	Delta
Omicron BA.1	Omicron BA.1
Omicron BA.1/BA.2	Omicron BA.1/Omicron BA.2
Omicron BA.4/BA.5	Omicron BA.5
Omicron BA.5	Omicron BA.5

Matches made for variant between progression studies and neutralisation titres. Note that for any row in right column with multiple entries, these were combined as the (unweighted) geometric mean of the titres for each entry.

Table F Time since last dose matches

Time since last dose (Clinical studies)	Time since last dose (Neutralisation database)
no information provided	1 month<br>2-6 months<br>6+ months
mean/median <= 30 days	1 month
mean/median 30 to < 180 days	2-6 months
mean/median >=180 days	6+ months
minimum 30 to < 180 days	2-6 months<br>6+ months
minimum >=180 days	6+ months
maximum <=30 days	1 month
maximum 30 to 180 days	1 month<br>2-6 months
range 14 to 55 days	1 month<br>2-6 months
range 56 to 83 days	2-6 months
range 91 to 180 days	2-6 months
range 84 to 111 days	2-6 months
range 112 to 139 days	2-6 months
range 7 to 179 days	1 month<br>2-6 months

Matches made for time since last dose between clinical studies and neutralisation titres. Note that for any row in right column with multiple entries, these were combined as the (unweighted) geometric mean of the titres for each entry.

Table G Intercept only model of vaccine protection from progression

Parameter	Beta	Std error	RR	95% CI for RR	p value
intercept	-1.322	0.114	0.27	0.21 to 0.33	<0.001
**Random effects**		τ^2	I^2		
level 1		0.0043			
level 2		0.2208	50.8%		
level 3		0.2093	48.2%		
Total		0.4344	99.0%		

Table H Vaccine protection from progression model comparison

Details of three models with the lowest AICc

Included covariates	AICc	I^2	Test for residual heterogeneity	Test of moderators (covariates)
vaccine regimen, age group, variant, time since last dose	190.65	Level 2: 28.5%<br>Level 3: 69.9%	QE(df = 119) = 5085.6<br>p < 0.001	QM(df = 17) = 157.9<br>p < 0.001
vaccine regimen, age group, variant	190.78	Level 2: 28.6%<br>Level 3: 69.9%	QE(df = 120) = 5372.1<br>p < 0.001	QM(df = 16) = 151.1<br>p < 0.001
vaccine regimen, age group, variant, time since last dose, endpoint	193.38	Level 2: 28.4%<br>Level 3: 70.1%	QE(df = 118) = 5081.2<br>p < 0.001	QM(df = 18) = 158.0<br>p < 0.001

Table I Multivariate model of vaccine protection from progression

Parameter		Beta	Std error	RR	95% CI for RR	p value^
vaccine Regimen						
	no vaccination	Ref.				<0.001
	Ad26.COV2.S primary	-0.602	0.227	0.55	0.35 to 0.85	
	AZD122 primary	-0.520	0.185	0.59	0.41 to 0.85	
	2x AZD122 + 1x mRNA	-1.210	0.189	0.30	0.21 to 0.43	
	BBIBP-CorV primary	-0.765	0.316	0.47	0.25 to 0.86	
	3x BNT162b2	-1.735	0.242	0.18	0.11 to 0.28	
	BNT162b2 primary	-1.108	0.202	0.33	0.22 to 0.49	
	4x combined mRNA	-1.437	0.359	0.24	0.12 to 0.48	
	3x combined mRNA	-1.075	0.170	0.34	0.24 to 0.48	
	combined mRNA primary	-0.809	0.173	0.45	0.32 to 0.62	
	3x CoronaVac	-1.721	0.293	0.18	0.10 to 0.32	
	CoronaVac primary	-1.022	0.251	0.36	0.22 to 0.59	
	2x CoronaVac + 1x BNT162b2	-1.712	0.300	0.18	0.10 to 0.33	
	CoronaVac/BBIBP-CorV primary	-1.319	0.877	0.27	0.05 to 1.49	
	mRNA-1273 primary	-1.076	0.330	0.34	0.18 to 0.65	
	Sputnik V primary	-0.703	0.387	0.49	0.23 to 1.06	
variant						
	Omicron	Ref.				
	pre-Omicron	-0.531	0.072	0.59	0.51 to 0.68	<0.001
age group						
	40-79 yrs	Ref.				
	<40 yrs	-0.410	0.162	0.66	0.48 to 0.91	0.013
time since last dose						
	per month	0.031	0.018	1.03	1.00 to 1.07	0.090
**Random effects**		τ^2	I^2			
level 1		0.0043				
level 2		0.0801	28.5%			
level 3		0.1964	69.9%			
Total		0.2808	98.5%			

^p values are from likelihood ratio tests comparing the full model with the model excluding the given covariate.

Table J Model with neutralising antibody titres as the only covariate

Parameter	Beta	Std error	RR	95%CI for RR	p value
intercept	-0.396	0.151	0.67	0.50 to 0.90	0.0088
$\log_{10}$(gmt)	-0.562	0.056	0.57	0.51 to 0.64	<0.001
**Random effects**	τ^2	I^2			
level 1	0.0043				
level 2	0.1041	27.0%			
level 3	0.2766	71.8%			
Total	0.3850	98.9%			

Table K Model comparison: neutralising antibodies plus covariates

Included covariates	RR per $\log_{10}$(gmt)	95%CI for RR	p value	Log likelihood	p value for LRT^
$\log_{10}$(gmt) [reference model]	0.57	0.51 to 0.64	<0.001	-86.83	
$\log_{10}$(gmt), variant	0.62	0.54 to 0.70	<0.001	-83.57	0.0107
$\log_{10}$(gmt), age group	0.57	0.51 to 0.64	<0.001	-83.94	0.0164
$\log_{10}$(gmt), regimen	0.46	0.38 to 0.57	<0.001	-75.74	0.0751
$\log_{10}$(gmt), TSLD#	0.57	0.50 to 0.65	<0.001	-86.82	0.9154

^p values are from likelihood ratio tests comparing the model with only neutralising antibodies to the model with the addition of the given covariate.
#Time since last dose

Table L Models including age group and variant as covariates

Parameter		Beta	Std error	RR	95% CI for RR	p value#
**Model 1: Neutralisation titre and variant**						
intercept		-0.386	0.147	0.68	0.51 to 0.91	0.0087
$\log_{10}$(gmt)		-0.485	0.062	0.62	0.54 to 0.70	<0.001
Variant						
	Omicron	Ref.				
	pre-Omicron	-0.227	0.088	0.80	0.67 to 0.95	0.0098
**Random effects**		τ^2	I^2			
level 1		0.0043				
level 2		0.0988	27.3%			
level 3		0.2592	71.6%			
Total		0.3623	98.8%			
**Model 2: Neutralisation titre and age**						
intercept		-0.172	0.177	0.84	0.59 to 1.19	0.33
$\log_{10}$(gmt)		-0.558	0.055	0.57	0.51 to 0.64	<0.001
age group						
	40-79 yrs	Ref.				
	<40 yrs	-0.425	0.174	0.65	0.46 to 0.92	0.015
**Random effects**		τ^2	I^2			
level 1		0.0043				
level 2		0.0976	24.7%			
level 3		0.2935	74.2%			
Total		0.3954	98.9%			
**Model 3: Neutralisation titre with variant and age**						
intercept		-0.177	0.173	0.84	0.60 to 1.18	0.31
$\log_{10}$(gmt)		-0.487	0.061	0.61	0.55 to 0.69	<0.001
Variant						
	Omicron	Ref.				
	pre-Omicron	-0.213	0.086	0.81	0.68 to 0.96	0.013
age group						
	40-79 yrs	Ref.				
	<40 yrs	-0.397	0.171	0.67	0.48 to 0.94	0.020
**Random effects**		τ^2	I^2			
level 1		0.0043				
level 2		0.0931	25.0%			
level 3		0.2758	73.9%			
Total		0.3732	98.9%			

#p values in this table are from Wald tests

Table M Sensitivity analyses

Parameter	Beta	Std error	RR	95% CI for RR	p value[#]
**Model 1: Death as clinical outcome**					
intercept	-0.275	0.247	0.76	0.47 to 1.23	0.27
log10(gmt)	-0.759	0.080	0.47	0.40 to 0.55	<0.001
**Random effects**	τ^2	I^2			
level 1	0.0083				
level 2	0.1155	26.3%			
level 3	0.3158	71.8%			
Total	0.4396	98.1%			
**Model 2: Excluding >10% hospitalisation rates**					
intercept	-0.361	0.153	0.70	0.52 to 0.94	0.018
log10(gmt)	-0.600	0.061	0.55	0.49 to 0.62	<0.001
**Random effects**	τ^2	I^2			
level 1	0.0050				
level 2	0.1084	35.2%			
level 3	0.1942	63.1%			
Total	0.3076	98.4%			
**Model 3: Excluding high (>10%) and unknown prior infection rates**					
intercept	-0.157	0.157	0.85	0.63 to 1.16	0.32
log10(gmt)	-0.522	0.063	0.59	0.52 to 0.67	<0.001
**Random effects**	τ^2	I^2			
level 1	0.0042				
level 2	0.0958	50.3%			
level 3	0.0905	47.5%			
Total	0.1905	97.8%			

[#] p values in this table are from Wald tests

Results for models using death as the endpoint for disease progression (Model 1), excluding studies with high (>10%) rates of hospitalisation (Model 2), and excluding studies with unknown or high (>10%) rates of prior infection.

Table N Variant investigations

Parameter		Beta	Std error	RR	95% CI for RR	p value[#]
**Model 1: Overlapping titre range**						
intercept		-0.141	0.329	0.87	0.46 to 1.65	0.67
log10(gmt)		-0.611	0.159	0.54	0.40 to 0.74	<0.001
Variant						
	Omicron	Ref.				
	pre-Omicron	-0.329	0.117	0.72	0.57 to 0.91	0.0049
**Random effects**		τ^2	I^2			
level 1		0.0042				
level 2		0.1128	30.2%			
level 3		0.2564	68.7%			
Total		0.3734	98.9%			
**Model 2: same study, any timing**						
intercept		-0.415	0.209	0.66	0.44 to 1.00	0.047
log10(gmt)		-0.394	0.070	0.67	0.59 to 0.77	<0.001
Variant						
	Omicron	Ref.				
	pre-Omicron	-0.291	0.088	0.75	0.63 to 0.89	<0.001
**Random effects**		τ^2	I^2			
level 1		0.0048				
level 2		0.0808	22.4%			
level 3		0.2744	76.2%			
Total		0.3600	98.7%			
**Model 3: same study, concurrent**						
intercept		-0.329	0.185	0.72	0.50 to 1.03	0.075
log10(gmt)		-0.406	0.074	0.67	0.58 to 0.77	<0.001
Variant						
	Omicron	Ref.				
	pre-Omicron	-0.360	0.095	0.70	0.58 to 0.84	<0.001
**Random effects**		τ^2	I^2			
level 1		0.0072				
level 2		0.0672	41.2%			
level 3		0.0886	54.4%			
Total		0.1630	95.6%			

[#] p values in this table are from Wald tests

Results for models investigating the effect of variant. **Model 1** Restricting analysis to only observations within the range of neutralising antibody titres common to both pre-Omicron and Omicron variants. **Model 2** Restricting analysis to only studies that included results for both pre-Omicron and Omicron variants. **Model 3** Restricting analysis to only studies that included concurrently circulating pre-Omicron and Omicron variants. In all cases, *neutralising antibody titres (GMT)* and *variant* are still significant co-variates.

Figures

Figure A PRISMA flow diagram of included studies

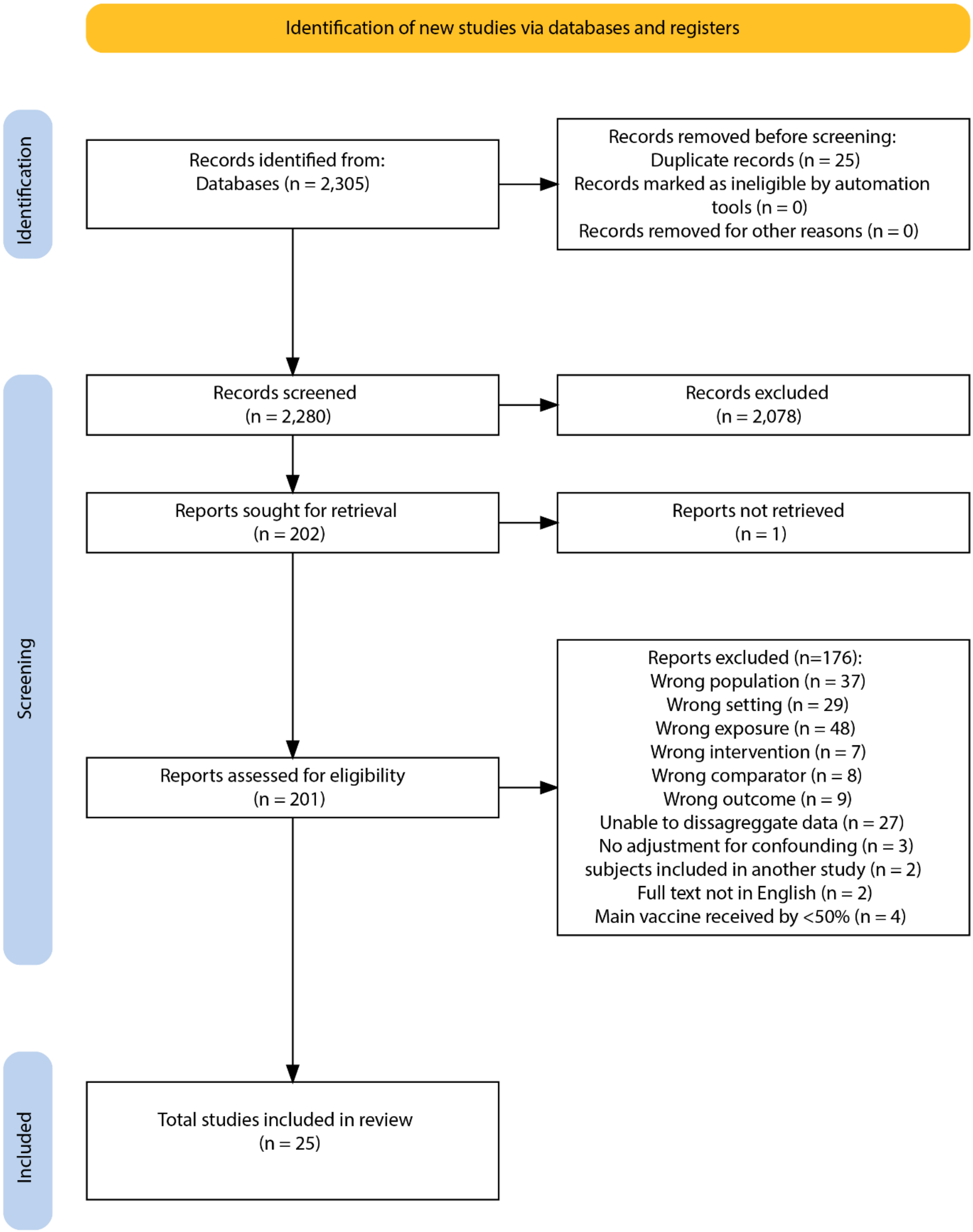

Figure B Forest plots for pre-Omicron variants

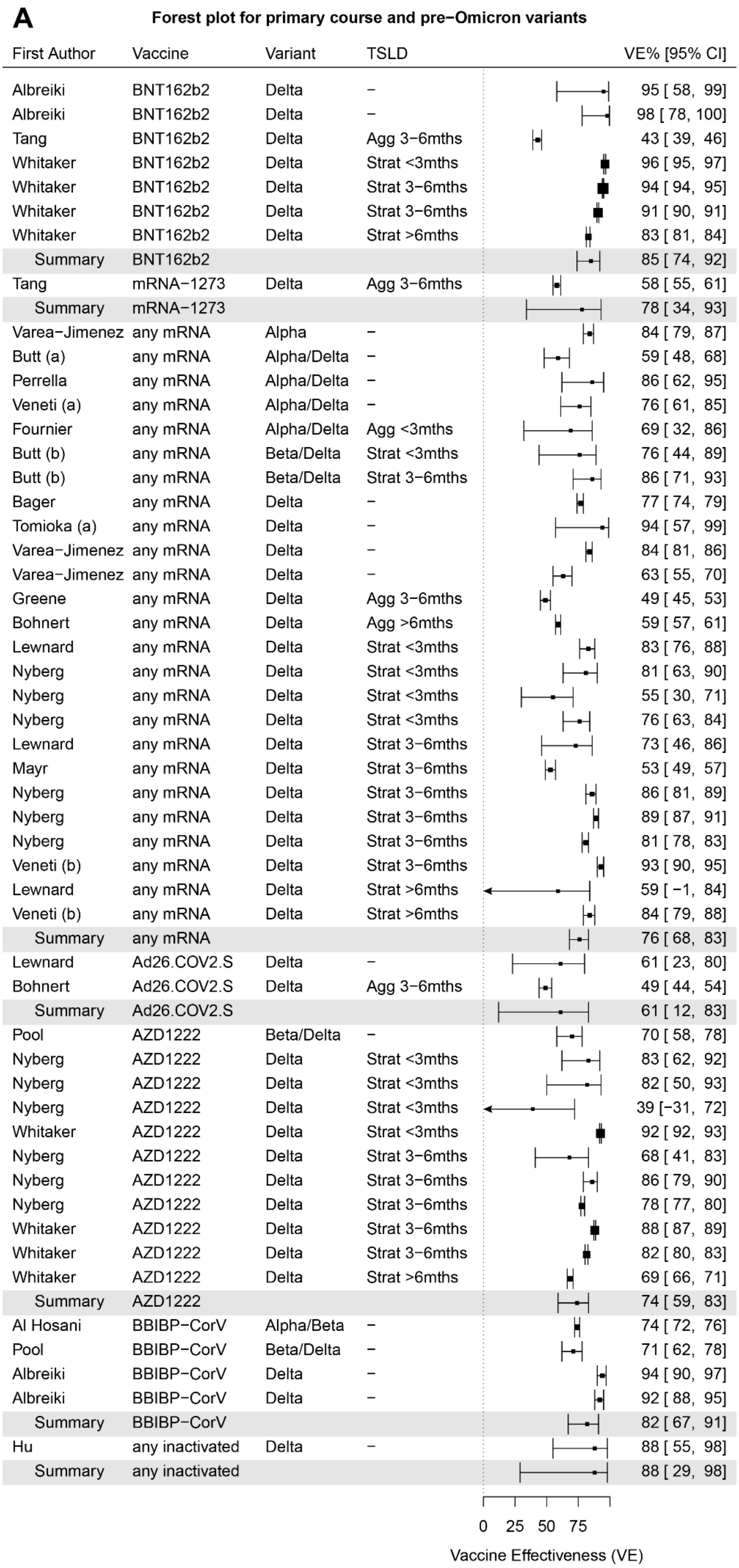

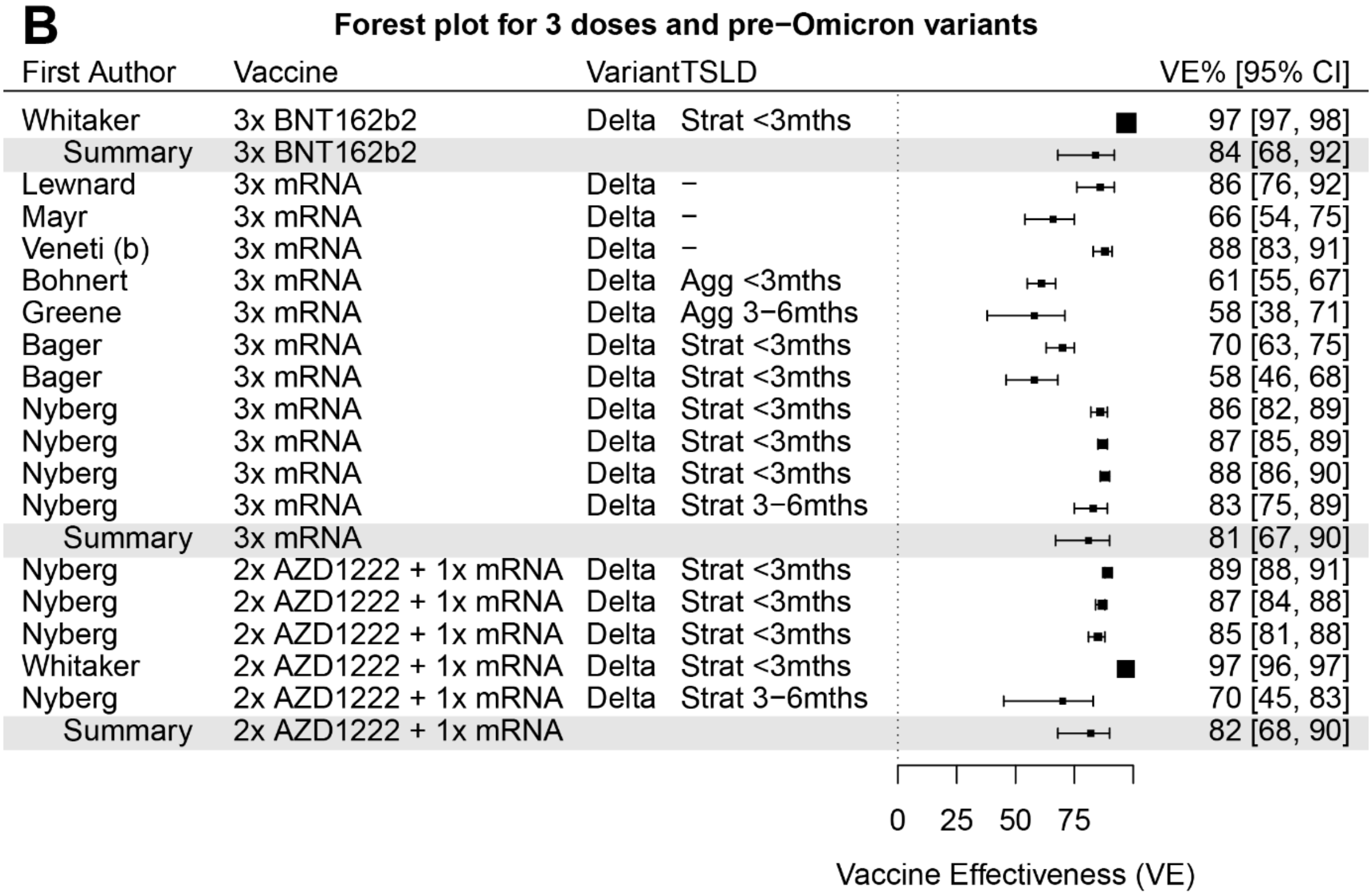

Forest plots showing vaccine effectiveness against progression to severe outcomes for any pre-Omicron variant, with (A) a complete primary course and (B) complete primary plus one more dose. Any mRNA refers to cohorts who had received a primary course of either BNT162b2 or mRNA-1273. Any inactivated refers to cohorts who had received a primary course of either CoronaVac or BBIBP-CorV. AZD1222/mRNA refers to cohorts who received a primary course of AZD1222 with any mRNA third dose. TSLD is time since last dose, where "Agg" means a study where the time since last dose was reported as a mean or median for the entire cohort, "Strat" means a study where outcomes were reported stratified by time since last dose. Rows with the same first author, vaccine, variant and TSLD are due to results that were stratified by age (≤45yrs vs. >45yrs, Albreiki), cover two separate enrolment time periods (Varea-Jiminez), or where the stratification by TSLD did not align exactly (e.g. study stratified by month, reporting three separate VEs that are all <3 months) with the groupings shown (all other cases).

Figure C Forest plots for Omicron variants

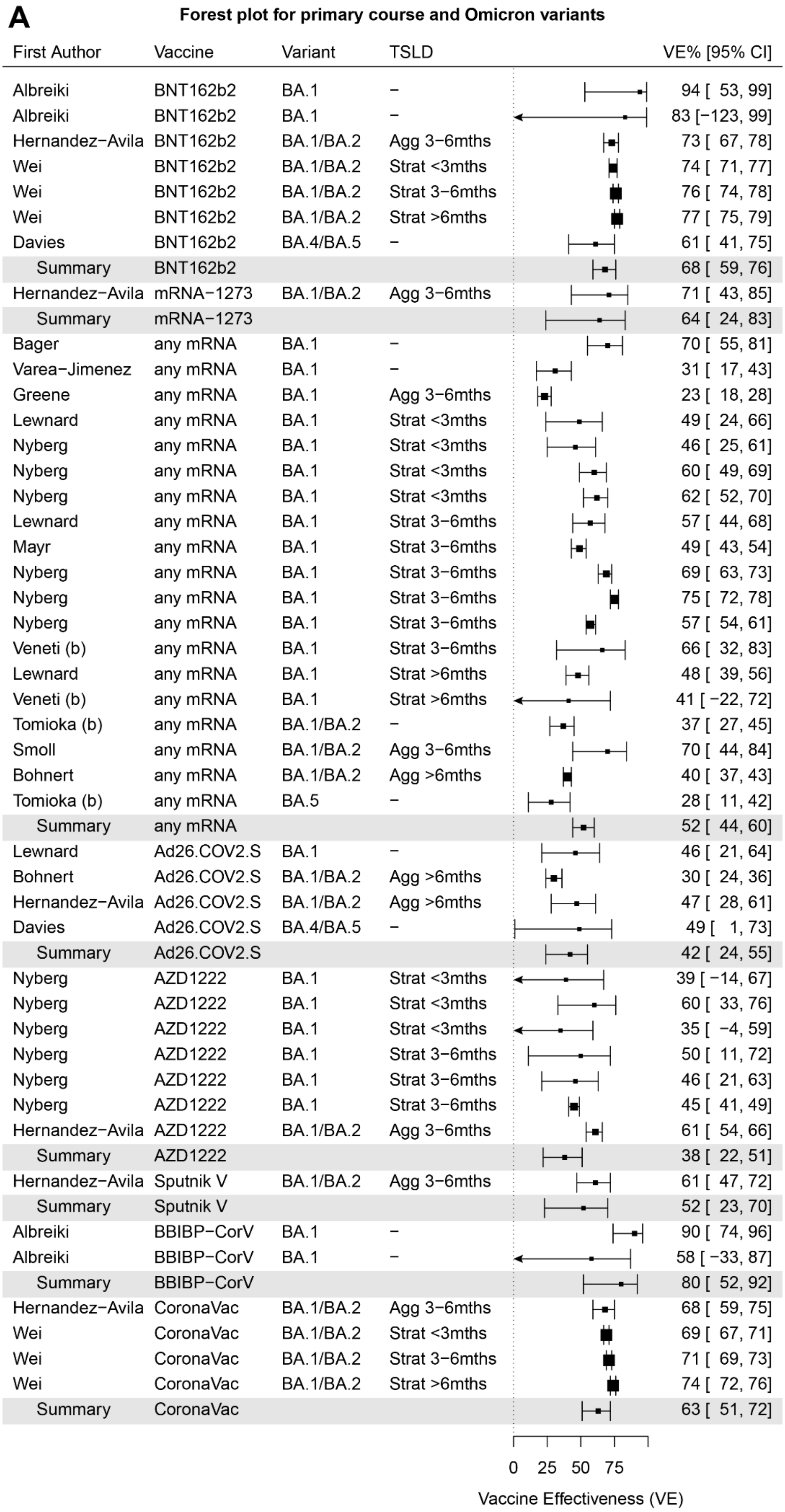

B

Forest plot for 3 or 4 doses and Omicron variants

First Author	Vaccine	Variant	TSLD	VE% [95% CI]
Wei	3x BNT162b2	BA.1/BA.2	Strat <3mths	84 [82, 86]
Wei	3x BNT162b2	BA.1/BA.2	Strat 3−6mths	85 [80, 89]
Davies	3x BNT162b2	BA.4/BA.5	−	80 [51, 92]
Summary	3x BNT162b2			84 [74, 90]
Bager	3x mRNA	BA.1	−	70 [43, 85]
Lewnard	3x mRNA	BA.1	−	57 [48, 65]
Mayr	3x mRNA	BA.1	−	74 [66, 80]
Veneti (b)	3x mRNA	BA.1	−	86 [69, 94]
Greene	3x mRNA	BA.1	Agg 3−6mths	63 [60, 65]
Nyberg	3x mRNA	BA.1	Strat <3mths	77 [74, 80]
Nyberg	3x mRNA	BA.1	Strat <3mths	74 [71, 77]
Nyberg	3x mRNA	BA.1	Strat <3mths	78 [76, 80]
Nyberg	3x mRNA	BA.1	Strat 3−6mths	81 [78, 83]
Tomioka (b)	3x mRNA	BA.1/BA.2	Agg <3mths	57 [49, 64]
Bohnert	3x mRNA	BA.1/BA.2	Agg 3−6mths	61 [59, 62]
Tomioka (b)	3x mRNA	BA.5	Agg 3−6mths	45 [36, 53]
Summary	3x mRNA			67 [60, 73]
Tomioka (b)	4x mRNA	BA.5	Agg <3mths	69 [62, 74]
Summary	4x mRNA			78 [68, 85]
Nyberg	2x AZD1222 + 1x mRNA	BA.1	Strat <3mths	71 [67, 74]
Nyberg	2x AZD1222 + 1x mRNA	BA.1	Strat <3mths	78 [76, 80]
Nyberg	2x AZD1222 + 1x mRNA	BA.1	Strat <3mths	79 [76, 81]
Nyberg	2x AZD1222 + 1x mRNA	BA.1	Strat 3−6mths	77 [72, 81]
Summary	2x AZD1222 + 1x mRNA			66 [57, 74]
Wei	3x CoronaVac	BA.1/BA.2	Strat <3mths	84 [82, 86]
Wei	3x CoronaVac	BA.1/BA.2	Strat 3−6mths	88 [86, 90]
Summary	3x CoronaVac			85 [77, 91]
Wei	2x CVac + 1x BNT162b2	BA.1/BA.2	Strat <3mths	86 [83, 89]
Wei	2x CVac + 1x BNT162b2	BA.1/BA.2	Strat 3−6mths	86 [82, 90]
Summary	2x CVac + 1x BNT162b2			85 [76, 91]

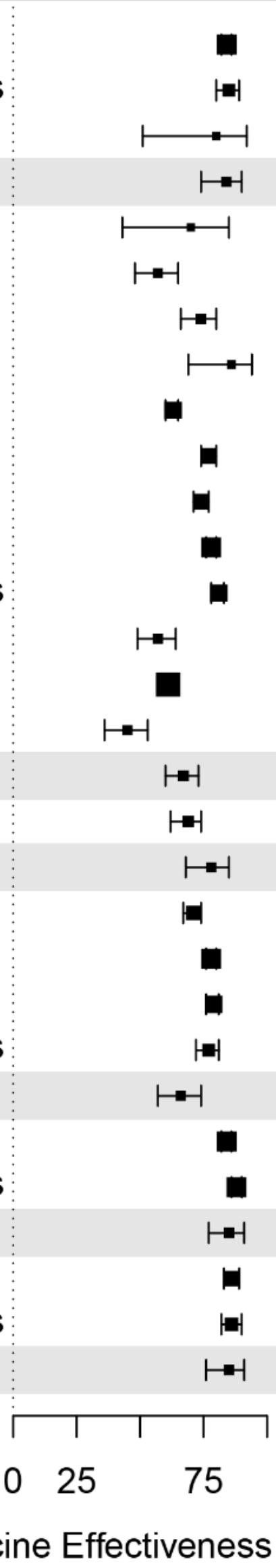

Forest plots showing vaccine effectiveness against progression to severe outcomes for any Omicron variant, with (A) a complete primary course and (B) complete primary plus one or two more doses. Any mRNA refers to cohorts who had received a primary course of either BNT162b2 or mRNA-1273. AZD1222/mRNA refers to cohorts who received a primary course of AZD1222 with any mRNA third dose. CVac/BNT162b2 refers to cohorts who received CoronaVac primary course and BNT162b2 third dose. TSLD is time since last dose, where “Agg” means a study where the time since last dose was reported as a mean or median for the entire cohort, “Strat” means a study where outcomes were reported stratified by time since last dose. Rows with the same first author, vaccine, variant and TSLD are due to results that were stratified by age (≤45yrs vs. >45yrs, Albreiki), or where the stratification by TSLD did not align exactly (e.g. study stratified by month, reporting three separate VEs that are all <3 months) with the groupings shown (all other cases).

Figure D Vaccine protection against progression by age group

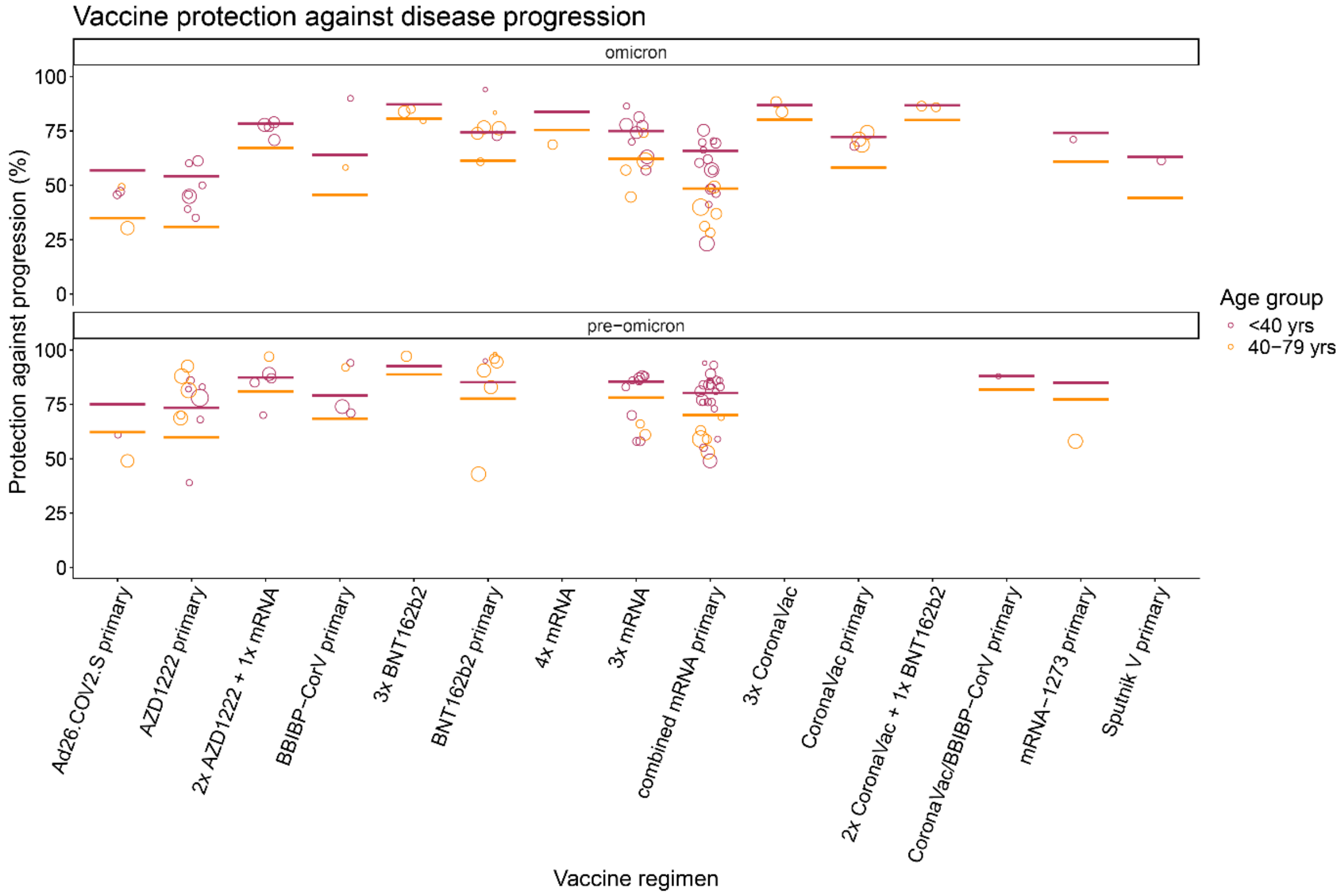

Circles show reported protection against progression (calculated as 100 x (1-RR)), with size proportional to inverse variance of reported result. Lines show model fit for each combination of regimen, variant, and age group.

Figure E Neutralisation titres by vaccine regimen and variant

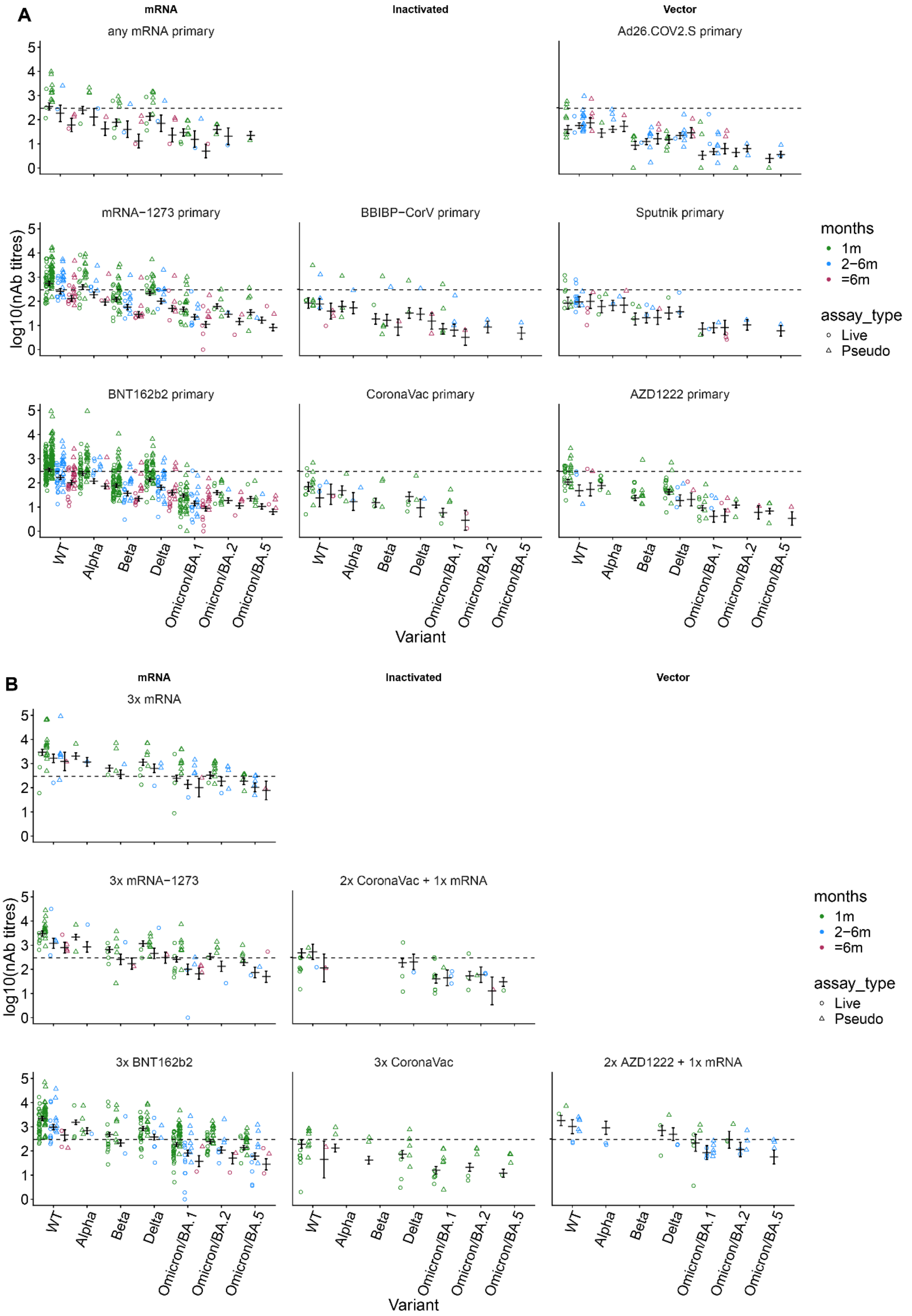

Neutralisation titres (geometric mean titre) by vaccine regimen and variant for (A) primary course and (B) 3 doses. Points are obtained from the Stanford database of neutralisation titres with circles used for live virus assay results and triangles used for pseudovirus assay results. Colours show the time since last dose category (from the Stanford database). The model fit and 95% CI for the given variant and vaccine regimen is shown in black (for live virus assays). The horizontal dashed line at 2.476 (corresponding to a geometric mean titre of $10^{2.48}$ = 299) shows the ($\log_{10}$) geometric mean neutralising titre against the ancestral strain among convalescent individuals who experienced ancestral SARS-CoV-2 infection (obtained from the Stanford database – Susceptability summaries Table 2 on the website), and was used to normalise GMTs to a fold-convalescent scale.

Figure F Alternate models

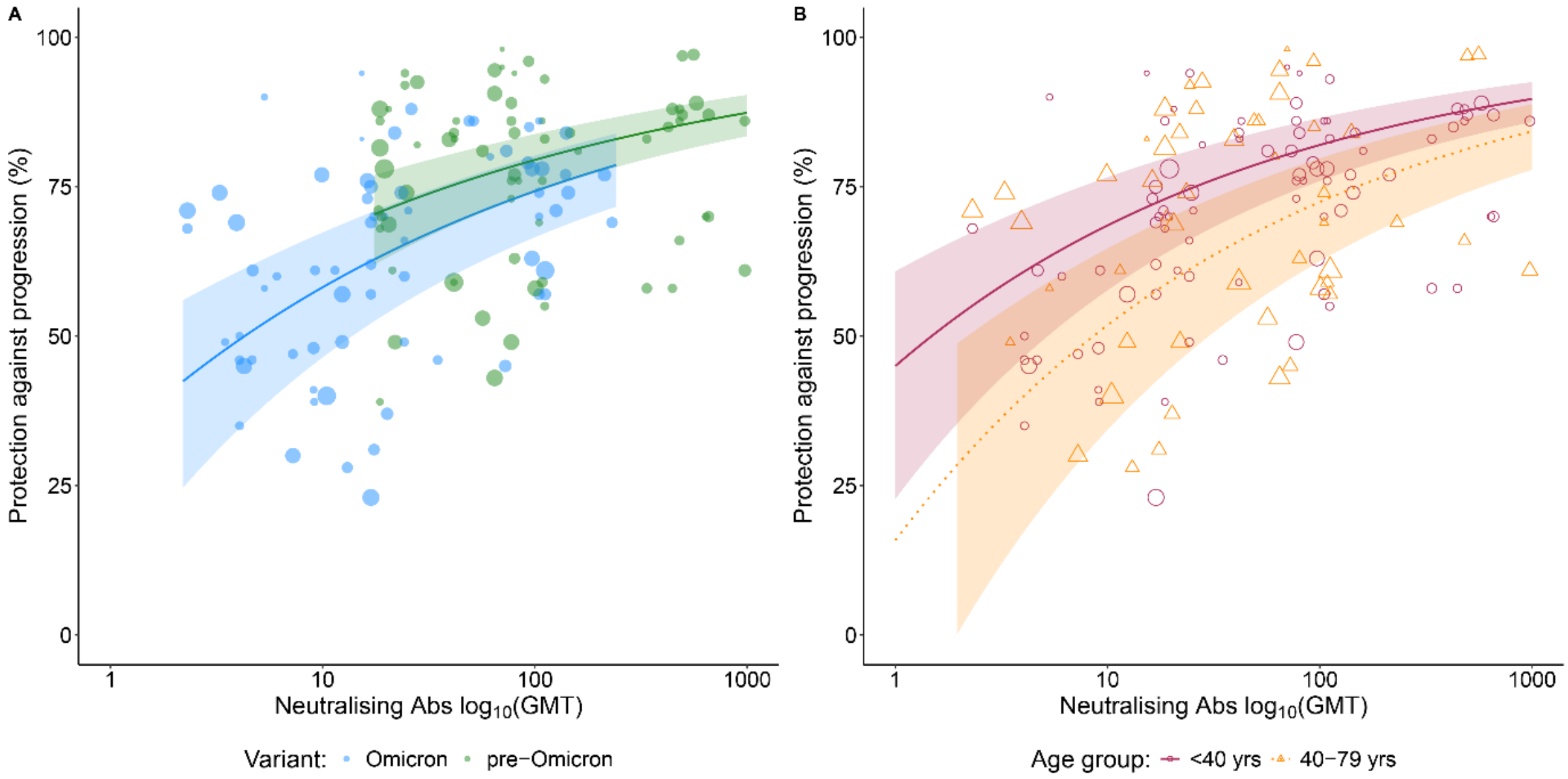

Model including neutralisation titre and variant (A), showing lower protection for Omicron variants for a given titre. Model including neutralisation titre and age group (B), showing lower protection in the older age group for a given titre. Protection against progression is calculated as 100 x (1 – aRR). Shaded regions show 95% confidence intervals.

Figure G Influential observations

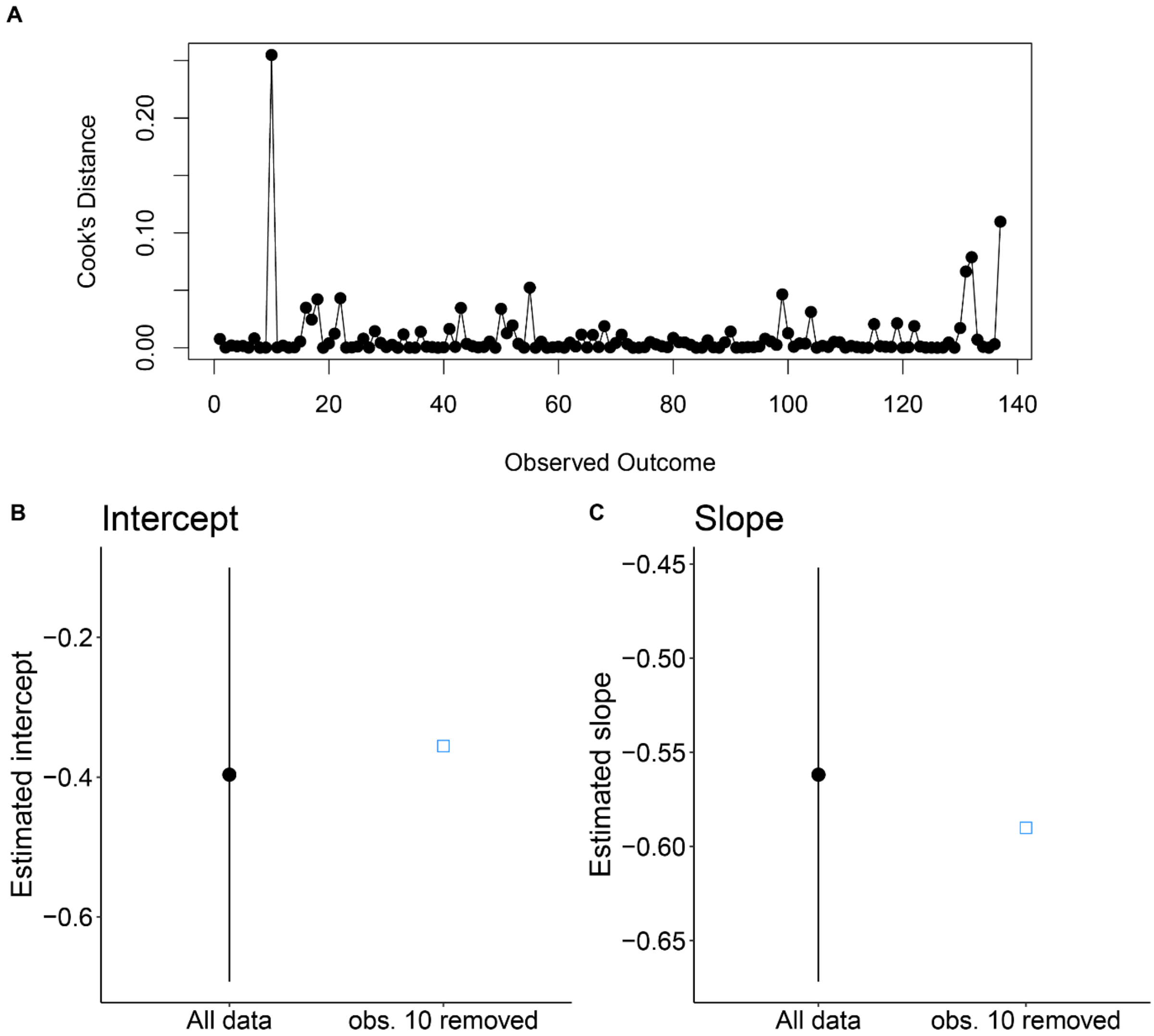

Cooks distance showing observation 10 as the most influential (A), with effects of removing this observation on estimated intercept (B) and slope (C). Black point and line shows estimate and 95% CI with all data, blue square shows esimate when model is refit excluding influential observation.

Figure H Leave-one-study-out analysis

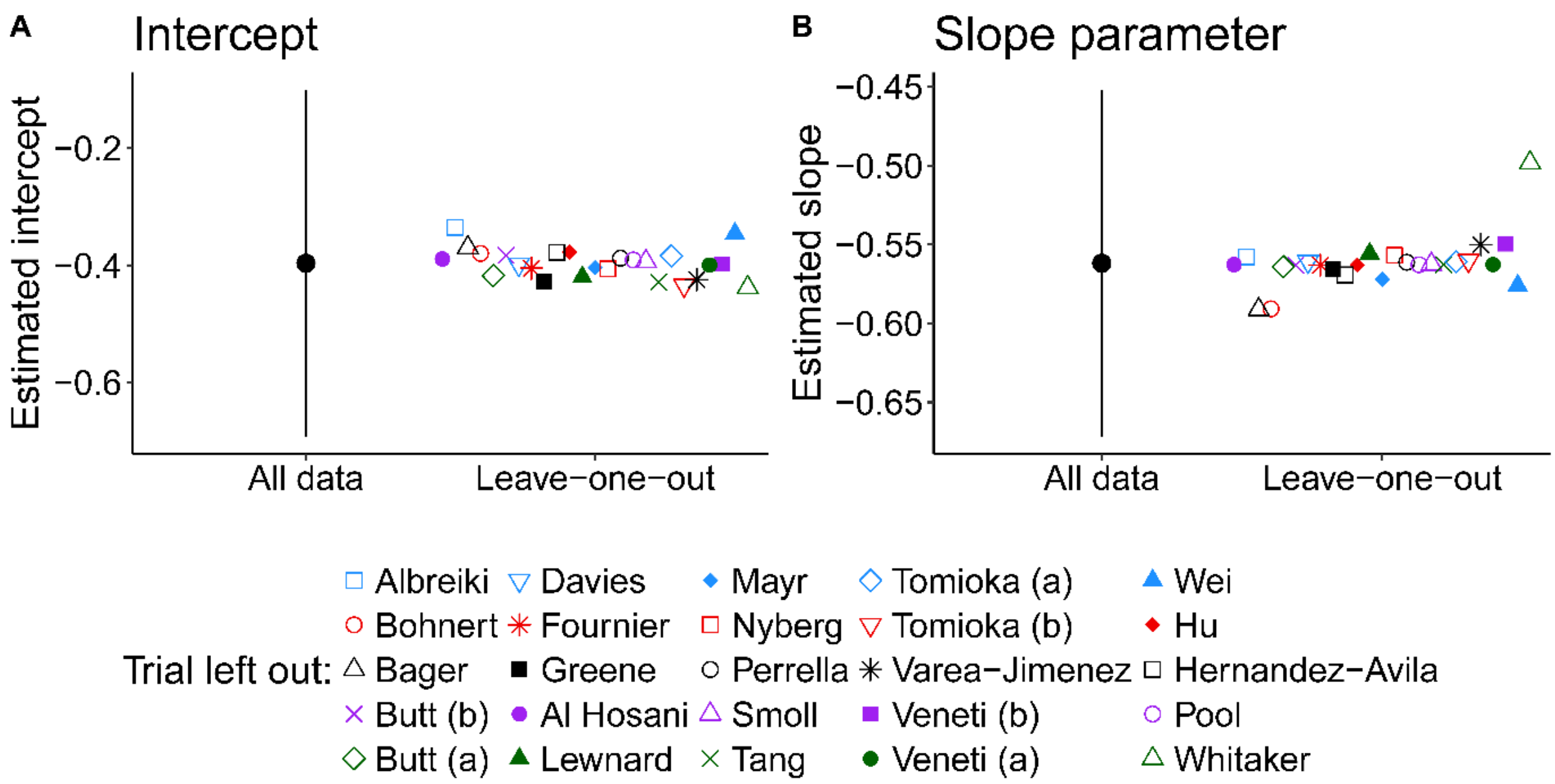

All data, shown as black circle and line, is the estimate and 95% CI for intercept (A) and slope (B) with all studies included, while the leave-one-out section shows the same estimates from the model refit excluding the named study.

Figure I Funnel plot

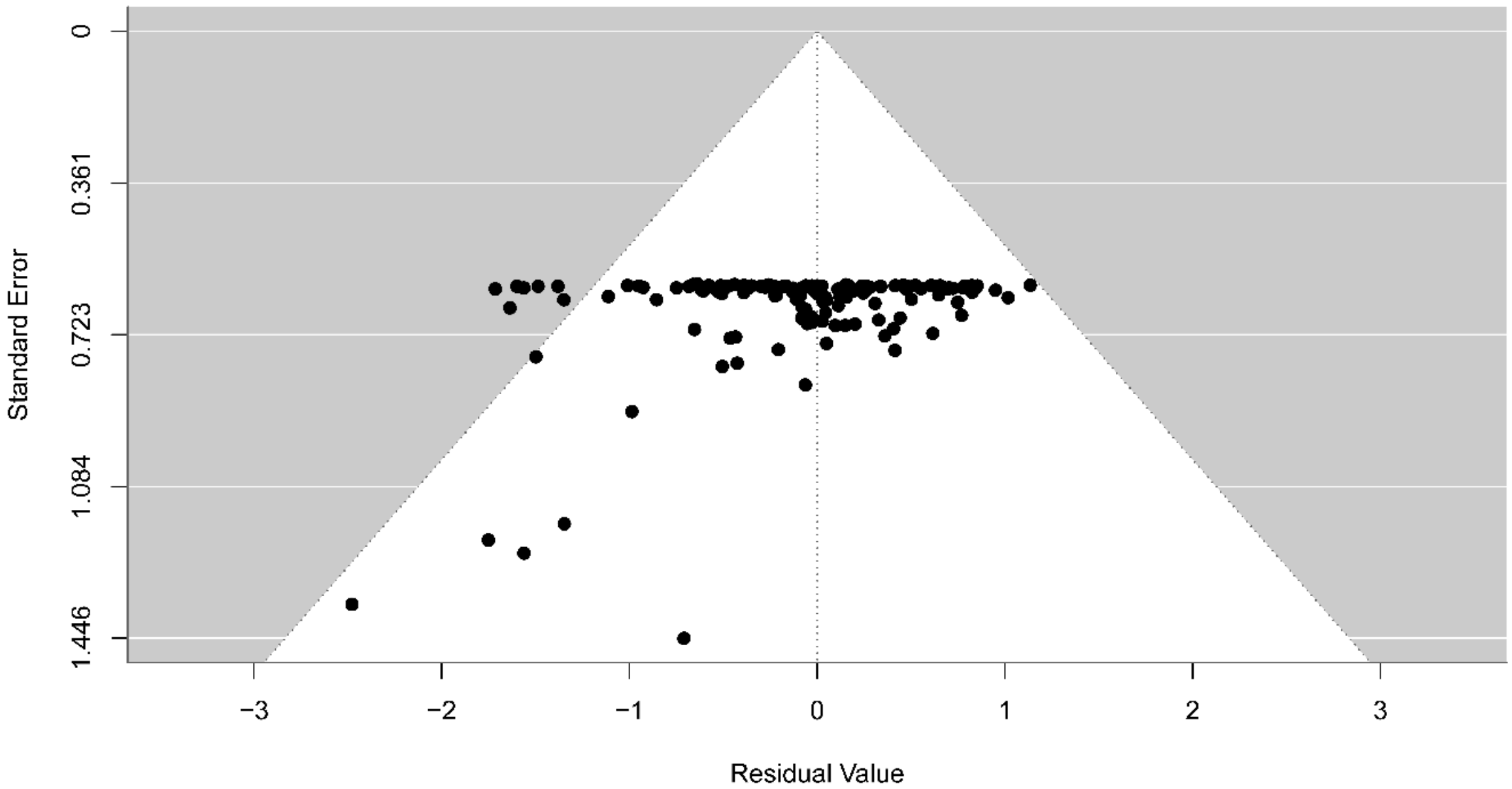

Funnel plot for the statistical model in Figure 3 and Supp. Table J, with a covariate of log_{10}(GMT) and nested random effects for observations within studies.

Figure J Effect of timing difference between antibody treatment and vaccination

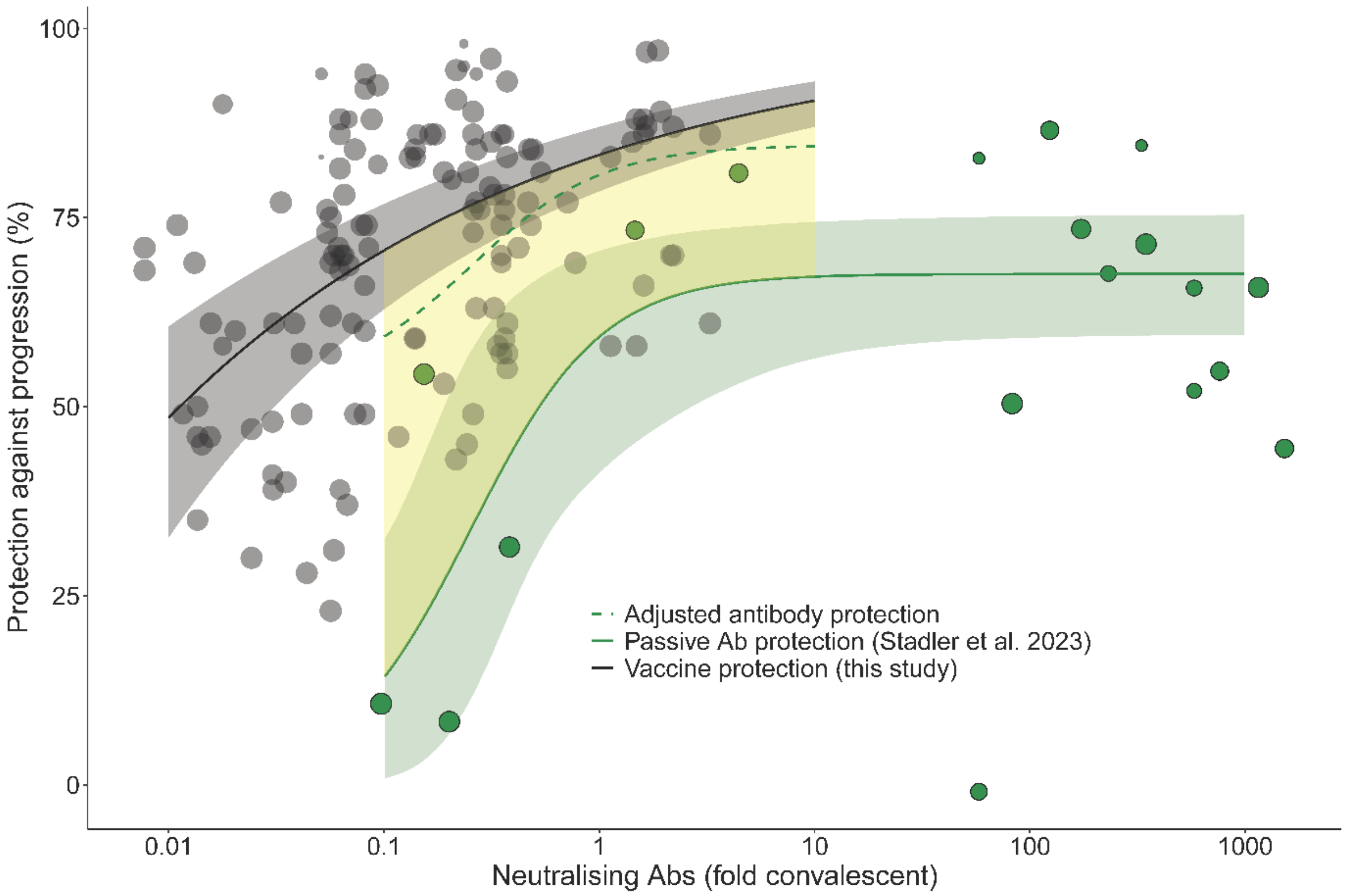

Estimated effect of timing difference due to therapeutic antibodies being administered post-symptom onset, while vaccine-induced antibodies are present at time of breakthrough infection. The black line shows the meta-regression model developed here (from Figure 3) with the shaded region showing the 95% CI (uncertainty in the estimate of the mean effect size pooled across studies). The green line shows the previously determined relationship between neutralisation titres and protection from progression for therapeutically administered antibodies (from Figure 4) [12]. For the range of neutralisation titres that is common to both vaccination and therapeutic antibodies, the protection gap is shown in yellow, while the green dashed line shows the effect of accounting for the previously determined lower risk of progression to severe outcomes if antibodies were administered at time of symptom onset instead of an average of 4.2 days later [12]. Protection against progression is calculated as 100 x (1 – aRR), shaded regions are 95% confidence intervals.

References

1. Butt AA, Yan P, Shaikh OS, Mayr FB, Omer SB. Rate and Risk Factors for Severe/Critical Disease Among Fully Vaccinated Persons With Breakthrough Severe Acute Respiratory Syndrome Coronavirus 2 (SARS-CoV-2) Infection in a High-Risk National Population. Clinical Infectious Diseases. 2021;75(1):e849-e56. doi: 10.1093/cid/ciab1023.
2. Perrella A, Bisogno M, D'Argenzio A, Trama U, Coscioni E, Orlando V. Risk of SARS-CoV-2 Infection Breakthrough among the Non-Vaccinated and Vaccinated Population in Italy: A Real-World Evidence Study Based on Big Data. Healthcare (Basel). 2022;10(6):1085. Epub 20220610. doi: 10.3390/healthcare10061085. PubMed PMID: 35742137; PubMed Central PMCID: PMCPMC9222607.
3. CDC. SARS-CoV-2 Variant Proportions [cited 2024 Sep]. Available from: https://data.cdc.gov/Laboratory-Surveillance/SARS-CoV-2-Variant-Proportions/jr58-6ysp/data_preview.
4. Albreiki M, Mousa M, Azman SK, Vurivi H, Alhalwachi Z, Alshehhi F, et al. Risk of hospitalization and vaccine effectiveness among COVID-19 patients in the UAE during the Delta and Omicron outbreaks. Front Immunol. 2023;14:1049393. Epub 20230213. doi: 10.3389/fimmu.2023.1049393. PubMed PMID: 36860855; PubMed Central PMCID: PMCPMC9969353.
5. Yin C, Hu B, Li K, Liu X, Wang S, He R, et al. Clinical characteristics and prognostic nomograms of 12555 non-severe COVID-19 cases with Omicron infection in Shanghai. BMC Infect Dis. 2023;23(1):606. Epub 20230916. doi: 10.1186/s12879-023-08582-5. PubMed PMID: 37716953; PubMed Central PMCID: PMCPMC10504722.
6. Tzou PL, Tao K, Pond SLK, Shafer RW. Coronavirus Resistance Database (CoV-RDB): SARS-CoV-2 susceptibility to monoclonal antibodies, convalescent plasma, and plasma from vaccinated persons. PLoS One. 2022;17(3):e0261045. Epub 20220309. doi: 10.1371/journal.pone.0261045. PubMed PMID: 35263335; PubMed Central PMCID: PMCPMC8906623.
7. Davies MA, Morden E, Rousseau P, Arendse J, Bam JL, Boloko L, et al. Outcomes of laboratory-confirmed SARS-CoV-2 infection during resurgence driven by Omicron lineages BA.4 and BA.5 compared with previous waves in the Western Cape Province, South Africa. Int J Infect Dis. 2023;127:63-8. Epub 20221124. doi: 10.1016/j.ijid.2022.11.024. PubMed PMID: 36436752; PubMed Central PMCID: PMCPMC9686046.
8. Ciabattini A, Nardini C, Santoro F, Garagnani P, Franceschi C, Medaglini D. Vaccination in the elderly: The challenge of immune changes with aging. Semin Immunol. 2018;40:83-94. doi: 10.1016/j.smim.2018.10.010. PubMed PMID: 30501873.
9. Nyberg T, Ferguson NM, Nash SG, Webster HH, Flaxman S, Andrews N, et al. Comparative analysis of the risks of hospitalisation and death associated with SARS-CoV-2 omicron (B.1.1.529) and delta (B.1.617.2) variants in England: a cohort study. Lancet. 2022;399(10332):1303-12. Epub 20220316. doi: 10.1016/S0140-6736(22)00462-7. PubMed PMID: 35305296; PubMed Central PMCID: PMCPMC8926413.
10. Cummings P. The Relative Merits of Risk Ratios and Odds Ratios. Archives of Pediatrics & Adolescent Medicine. 2009;163(5):438-45. doi: 10.1001/archpediatrics.2009.31.
11. Khoury DS, Cromer D, Reynaldi A, Schlub TE, Wheatley AK, Juno JA, et al. Neutralizing antibody levels are highly predictive of immune protection from symptomatic SARS-CoV-2 infection. Nature Medicine. 2021;27(7):1205-11. doi: 10.1038/s41591-021-01377-8.
12. Stadler E, Chai KL, Schlub TE, Cromer D, Khan SR, Polizzotto MN, et al. Determinants of passive antibody efficacy in SARS-CoV-2 infection: a systematic review and meta-analysis. Lancet Microbe. 2023;4(11):e883-e92. doi: 10.1016/S2666-5247(23)00194-5. PubMed PMID: 37924835.
13. Ismail AlHosani F, Eduardo Stanciole A, Aden B, Timoshkin A, Najim O, Abbas Zaher W, et al. Impact of the Sinopharm's BBIBP-CorV vaccine in preventing hospital admissions and death in infected vaccinees: Results from a retrospective study in the emirate of Abu Dhabi, United Arab Emirates (UAE). Vaccine. 2022;40(13):2003-10. Epub 20220219. doi:

10.1016/j.vaccine.2022.02.039. PubMed PMID: 35193793; PubMed Central PMCID: PMCPMC8857641.
14. Bager P, Wohlfahrt J, Bhatt S, Stegger M, Legarth R, Møller CH, et al. Risk of hospitalisation associated with infection with SARS-CoV-2 omicron variant versus delta variant in Denmark: an observational cohort study. Lancet Infect Dis. 2022;22(7):967-76. Epub 20220422. doi: 10.1016/s1473-3099(22)00154-2. PubMed PMID: 35468331; PubMed Central PMCID: PMCPMC9033212.
15. Bohnert AS, Kumbier K, Rowneki M, Gupta A, Bajema K, Hynes DM, et al. Adverse outcomes of SARS-CoV-2 infection with delta and omicron variants in vaccinated versus unvaccinated US veterans: retrospective cohort study. BMJ. 2023;381:e074521. doi: 10.1136/bmj-2022-074521.
16. Butt AA, Dargham SR, Chemaitelly H, Al Khal A, Tang P, Hasan MR, et al. Severity of Illness in Persons Infected With the SARS-CoV-2 Delta Variant vs Beta Variant in Qatar. JAMA Intern Med. 2022;182(2):197-205. doi: 10.1001/jamainternmed.2021.7949. PubMed PMID: 34935861; PubMed Central PMCID: PMCPMC8696690.
17. Fournier PE, Houhamdi L, Colson P, Cortaredona S, Delorme L, Cassagne C, et al. SARS-CoV-2 Vaccination and Protection Against Clinical Disease: A Retrospective Study, Bouches-du-Rhône District, Southern France, 2021. Front Microbiol. 2021;12:796807. Epub 20220118. doi: 10.3389/fmicb.2021.796807. PubMed PMID: 35116013; PubMed Central PMCID: PMCPMC8803903.
18. Greene SK, Levin-Rector A, Kyaw NTT, Luoma E, Amin H, McGibbon E, et al. Comparative hospitalization risk for SARS-CoV-2 Omicron and Delta variant infections, by variant predominance periods and patient-level sequencing results, New York City, August 2021-January 2022. Influenza Other Respir Viruses. 2023;17(1):e13062. Epub 20221031. doi: 10.1111/irv.13062. PubMed PMID: 36317297; PubMed Central PMCID: PMCPMC9835408.
19. Hu Z, Tao B, Li Z, Song Y, Yi C, Li J, et al. Effectiveness of inactivated COVID-19 vaccines against severe illness in B.1.617.2 (Delta) variant-infected patients in Jiangsu, China. Int J Infect Dis. 2022;116:204-9. Epub 20220119. doi: 10.1016/j.ijid.2022.01.030. PubMed PMID: 35065255; PubMed Central PMCID: PMCPMC8769614.
20. Lewnard JA, Hong VX, Patel MM, Kahn R, Lipsitch M, Tartof SY. Clinical outcomes associated with SARS-CoV-2 Omicron (B.1.1.529) variant and BA.1/BA.1.1 or BA.2 subvariant infection in Southern California. Nat Med. 2022;28(9):1933-43. Epub 20220608. doi: 10.1038/s41591-022-01887-z. PubMed PMID: 35675841; PubMed Central PMCID: PMCPMC10208005.
21. Mayr FB, Talisa VB, Castro AD, Shaikh OS, Omer SB, Butt AA. COVID-19 disease severity in US Veterans infected during Omicron and Delta variant predominant periods. Nature Communications. 2022;13(1):3647. doi: 10.1038/s41467-022-31402-4.
22. Smoll NR, Al Imam MH, Shulz C, Booy R, Khandaker G. The effectiveness of vaccination for preventing hospitalisation with COVID-19 in regional Queensland: a data linkage study. Med J Aust. 2023;219(4):162-5. Epub 20230703. doi: 10.5694/mja2.52019. PubMed PMID: 37400415.
23. Tang F, Hammel IS, Andrew MK, Ruiz JG. COVID-19 mRNA vaccine effectiveness against hospitalisation and death in veterans according to frailty status during the SARS-CoV-2 delta (B.1.617.2) variant surge in the USA: a retrospective cohort study. The Lancet Healthy Longevity. 2022;3(9):e589-e98. doi: 10.1016/S2666-7568(22)00166-0.
24. Tomioka K, Uno K, Yamada M. Association between vaccination status and COVID-19-related health outcomes among community-dwelling COVID-19 patients in Nara, Japan. Environ Health Prev Med. 2023;28:7. doi: 10.1265/ehpm.22-00199. PubMed PMID: 36682815; PubMed Central PMCID: PMCPMC9884564.
25. Tomioka K, Uno K, Yamada M. Association between vaccination status and severe health consequences among community-dwelling COVID-19 patients during Omicron BA.1/BA.2 and BA.5-predominant periods in Japan. Environ Health Prev Med. 2023;28:35. doi: 10.1265/ehpm.23-00061. PubMed PMID: 37286499; PubMed Central PMCID: PMCPMC10287986.

26. Varea-Jiménez E, Aznar Cano E, Vega-Piris L, Martínez Sánchez EV, Mazagatos C, García San Miguel Rodríguez-Alarcón L, et al. Comparative severity of COVID-19 cases caused by Alpha, Delta or Omicron SARS-CoV-2 variants and its association with vaccination. Enferm Infecc Microbiol Clin (Engl Ed). 2024;42(4):187-94. Epub 20230201. doi: 10.1016/j.eimce.2022.11.021. PubMed PMID: 36737369; PubMed Central PMCID: PMCPMC9890374.

27. Veneti L, Valcarcel Salamanca B, Seppala E, Starrfelt J, Storm ML, Bragstad K, et al. No difference in risk of hospitalization between reported cases of the SARS-CoV-2 Delta variant and Alpha variant in Norway. Int J Infect Dis. 2022;115:178-84. Epub 20211211. doi: 10.1016/j.ijid.2021.12.321. PubMed PMID: 34902584; PubMed Central PMCID: PMCPMC8664610.

28. Veneti L, Bøås H, Bråthen Kristoffersen A, Stålcrantz J, Bragstad K, Hungnes O, et al. Reduced risk of hospitalisation among reported COVID-19 cases infected with the SARS-CoV-2 Omicron BA.1 variant compared with the Delta variant, Norway, December 2021 to January 2022. Euro Surveill. 2022;27(4):2200077. doi: 10.2807/1560-7917.Es.2022.27.4.2200077. PubMed PMID: 35086614; PubMed Central PMCID: PMCPMC8796289.

29. Wei Y, Jia KM, Zhao S, Hung CT, Mok CKP, Poon PKM, et al. Estimation of Vaccine Effectiveness of CoronaVac and BNT162b2 Against Severe Outcomes Over Time Among Patients With SARS-CoV-2 Omicron. JAMA Network Open. 2023;6(2):e2254777-e. doi: 10.1001/jamanetworkopen.2022.54777.

30. Hernández-Ávila M, Vieyra-Romero WI, Gutiérrez-Díaz HO, Zepeda-Tello R, Alpuche-Aranda C, Hernández-Ávila JE, et al. The Omicron wave in Mexico: vaccine protection against progression to severe Covid-19 in SARS-CoV-2-infected workers. Salud Publica Mex. 2023;66(1, ene-feb):85-94. Epub 20231208. doi: 10.21149/15125. PubMed PMID: 38065107.

31. Pool SNT, Shroff EH, Chetty A, Lewis L, Nonhlanhla YZ, Abdool Karim SS. Effectiveness of ChAdOx1 nCoV-19 and BBIBP-CorV vaccines against COVID-19-associated hospitalisation and death in the Seychelles infected adult population. PLoS One. 2024;19(4):e0299747. Epub 20240405. doi: 10.1371/journal.pone.0299747. PubMed PMID: 38578809; PubMed Central PMCID: PMCPMC10997067.

32. Whitaker HJ, Tsang RSM, Byford R, Aspden C, Button E, Sebastian Pillai P, et al. COVID-19 vaccine effectiveness against hospitalisation and death of people in clinical risk groups during the Delta variant period: English primary care network cohort study. J Infect. 2023;87(4):315-27. Epub 20230812. doi: 10.1016/j.jinf.2023.08.005. PubMed PMID: 37579793.
